# ROS-sensitive liposomal co-delivery of photosensitizer, factor Xa inhibitor, and PD-L1 blockade enhances photodynamic immunotherapy

**DOI:** 10.7150/thno.125408

**Published:** 2026-01-21

**Authors:** Yuhan Mai, Yanling Chen, Chao Li, Tongyao Wang, Shangli Ding, Hao Zhang, Haili Lin, Longguang Jiang, Cai Yuan, Xiaolei Zhou, Mingdong Huang, Peng Xu

**Affiliations:** 1 College of Chemistry, Fuzhou University, Fuzhou, Fujian 350116, China P.R.; 2 College of Biological Science and Engineering, Fuzhou University, Fuzhou, Fujian 350108, China P.R.; 3 Department of Pharmacy, The Peoples Hospital of Fujian Province, Fuzhou, Fujian 350004, China P.R.; 4 Fujian Key Laboratory of Marine Enzyme Engineering, Fuzhou University, Fuzhou, Fujian 350108, China P.R.

**Keywords:** photodynamic therapy, immunotherapy, anticoagulant therapy, liposome, photo-specific release

## Abstract

**Background:** Compared to the lymphodepleting chemotherapy and radiotherapy, photodynamic therapy (PDT) is an oncotherapeutic modality inherently stimulating immune responses by inducing immunogenic cell death (ICD). However, the immunosuppressive tumor microenvironment (TME) frequently attenuates PDT-elicited immune responses, limiting its efficacy in eradicating distant and metastatic tumor cells.

**Methods:** To maximize the immunotherapeutic efficacy of PDT, we developed a photodynamic immunotherapeutic liposomal nanoplatform (PDIT-liposome) integrating components targeting sequential stages of the antitumor immune response: 1) a phthalocyanine photosensitizer to induce ICD, 2) a factor Xa inhibitor (rivaroxaban) to promote T-cell priming, 3) and a program death-ligand 1 inhibitor to augment cytotoxic T lymphocyte (CTL) attack. To enable light-controlled drug release at tumor sites, the liposome was constructed with reactive oxygen species-sensitive phospholipids in response to the PDT effect.

**Results**: PDIT-liposomes were characterized via multiple physicochemical and optical evaluations. Comprehensive *in vitro* and *in vivo* investigations confirmed that PDIT-liposomes significantly enhanced antitumor efficacy compared to monotherapies and dual combinations. In a subcutaneous implantation tumor model, PDIT-liposome achieved a 91.7% antitumor rate compared to 21.83% (P-liposome), 46.78% (PD-liposome), and 51.08% (PR-liposome) (p < 0.001). Mechanistic analysis revealed enhanced dendritic cell maturation (8-fold increase in CD11c^+^ cells) and T-cell priming (2.3-fold increase in CD8^+^ T cells) in tumor-draining lymph nodes (TDLNs), and CTL-mediated cytotoxicity (5.4-fold increase in CD107a^+^ activated CTLs) in TME. Notably, PDIT therapy induced long-term immunological memory, which suppressed 90.68% tumor reoccurrence and metastasis.

**Conclusion:** This study presents a strategy to amplify PDT-elicited immunotherapeutic efficacy by synergizing agents targeting distinct stages of the immune response. It also theoretically validates the synergy of PDT, anticoagulation therapy, and immune checkpoint inhibition in cancer treatment.

## Introduction

Since the late 1970s, when hematoporphyrin derivatives (HpDs) were first tested for melanoma treatment, PDT has emerged as a promising oncotherapeutic strategy. The core mechanism relies on a unique laser-induced cytotoxicity against tumor cells via the generation of reactive oxygen species (ROS) 1. Initially, PDT was believed to exert effects primarily through the disruption of cellular redox homeostasis: ROS damage critical cellular components including phospholipid bilayer of biological membranes, ultimately inducing tumor cell apoptosis or necrosis 2. In addition to direct cytotoxicity, PDT also effectively obstructs tumor angiogenesis, because tumor-associated neovasculature lacks smooth muscle coverage, and is highly vulnerable to PDT-induced oxidative stress 3. Such an anti-angiogenetic effect impairs tumor's nutrient/oxygen supply and hinders its metastatic potential. Collectively, PDT exhibits a dual action modality targeting both tumor cells and TME.

Recent researches have unveiled the immunomodulatory effects of PDT 4. Beyond triggering intrinsic apoptotic pathways via mitochondrial disruption, PDT generates robust ROS disrupting cell membranes leading to the exposure of tumor-associated antigens (TAAs), including mutated KRAS and HER2, and the release of damage-associated molecular patterns (DAMPs), e.g., calreticulin (CRT) and high mobility group box 1 (HMGB1) 5. Such PDT-induced ICD activates both innate and adaptive anti-cancer immune responses, promoting the recruitment and infiltration of immune cells into the tumor tissues 6. Notably, this immunostimulatory property distinguishes PDT from the immunosuppressive chemotherapy and radiotherapy, both of which compromise myeloid and immune functions 7. PDT-induced ICD is expected to promote achieving a long-term anti-tumor immunity overcoming the limitation of lacking systemic effects and persistence in traditional PDT 1. Additionally, PDT has also been reported to reprogram the “cold tumor” microenvironment to “hot tumor” phenotype by augmenting immune cell infiltration, and consequently potentiating the efficacy of immunotherapy 8. Based on these insights, Kobayashi and colleagues from the US National Institutes of Health (NIH) proposed a concept termed “Near Infrared Photoimmunotherapy (NIR-PIT)”. They developed a target-specific photosensitizer, RM-1929, by conjugating a phthalocyanine photosensitizer to an epidermal growth factor receptor (EGFR) monoclonal antibody 9. RM-1929 has demonstrated promising therapeutic efficacy in the recently finished Phase 1/2a clinical trials against head and neck squamous cell carcinoma 10. However, as an EGFR-targeted antibody-drug conjugate (ADC), RM-1929 has two key limitations: First, it can only precisely recognize EGFR on the surface of tumor cells and cannot overcome the immunosuppressive state of the tumor microenvironment; Second, RM-1929 is a fully water-soluble photosensitizer that struggles to penetrate cell membranes to destroy tumor cell structures from within. It can only cause localized damage to tumor cell membranes and exhibits lower phototoxicity compared to lipid-soluble photosensitizers, which can enter cells.

Moreover, the efficacy of such photodynamic immunotherapy (PDIT) is limited, primarily due to the presence of a TME. The immune response induced by PDT alone is often suppressed, manifesting in three key aspects: First, regulatory T cells (Treg) and tumor-associated macrophages (TAMs) largely accumulate in TME, secreting anti-inflammatory cytokines (e.g., IL-10, TGF-β, VEGF) that promote angiogenesis and downregulate dendritic cell (DC) maturation and T-cell activation 11. Second, myeloid-derived suppressor cells (MDSCs) further deplete essential amino acids (e.g., arginine) required for T-cell activation leading to T-cell exhaustion 12. Third, tumor cells overexpress Programmed Death-Ligand 1 (PD-L1), which binds to Programmed Death-1 (PD-1) receptors on T-cells triggering immunosuppressive signaling that drive T-cell dysfunction, exhaustion, and apoptosis 13. Given these TME-imposed immune-suppressive barriers, combining PDT with immunotherapy has emerged as a promising avenue to enhance anti-cancer efficacy 14. Although synergistic regimens involving immune checkpoint inhibitors have achieved encouraging results by enhancing the CTL attack, critical bottlenecks persist: antigen presentation by DCs and subsequent effective T-cell priming in both the TME and TDLNs remain impaired, which still undermines the full potential of PDT-induced adaptive immune responses 15, 16.

The interplay between the coagulation system and immune system has recently emerged as a critical area of cancer research. Clinical studies have revealed that upregulated coagulation factors, such as factor VIII (FVIII) and von Willebrand factor (vWF), correlate with poor prognosis and increased mortality in cancer patients 17. Additionally, platelets, thrombin, and factor Xa (FXa) have been implicated in various processes of tumor immune escape. For instance, upon contact with circulating tumor cells (CTCs), platelets become activated and form a protective coating around CTCs, creating an initial metastatic niche that shields CTCs from immune recognition 18, 19. Besides, platelet α-granules release various growth factors, including platelet-derived growth factor (PDGF) and vascular endothelial growth factor (VEGF), which drive tumor cell proliferation and angiogenesis within the TME 20. Additionally, tissue factor (TF) has been found to be overexpressed on triple-negative breast cancer cells and facilitate immune evasion by impeding T-cell infiltration and effector function 21. Intervention with TF signaling has been found to suppress early tumor progression in various cancer models 22-25. Furthermore, thrombin has been reported to promote tumor progression via proteolytic cleavage of glycoprotein A repetitions predominant (GARP), which results in the liberation of active TGF-β1 26. Inhibition of thrombin obliterates TGF-β1 maturation and reprograms the TME to favorable antitumor immunity. On top of these, coagulation factor X (FX), secreted by monocytes, macrophages and immunosuppressive neutrophils in the TME, plays a crucial role in driving immune evasion 27, 28. Myeloid cell-derived FXa activates protease-activated receptor 2 (PAR) signaling, inducing M2 polarization of TAMs and impairing antigen presentation of DCs. Notably, inhibition of FXa by rivaroxaban reprograms TAMs and enhances DCs and CTLs infiltration into tumor tissues, and therefore suppressing tumor growth and metastasis *in vivo* 27. More importantly, combining anticoagulant therapies (thrombin or FXa inhibitors) with immune checkpoint inhibitors has demonstrated synergistic effects improving overall response to cancer therapies 26, 27. Collectively, these studies highlight the coagulation system as potential therapeutic target for antitumor immunotherapy.

**Scheme 1:**
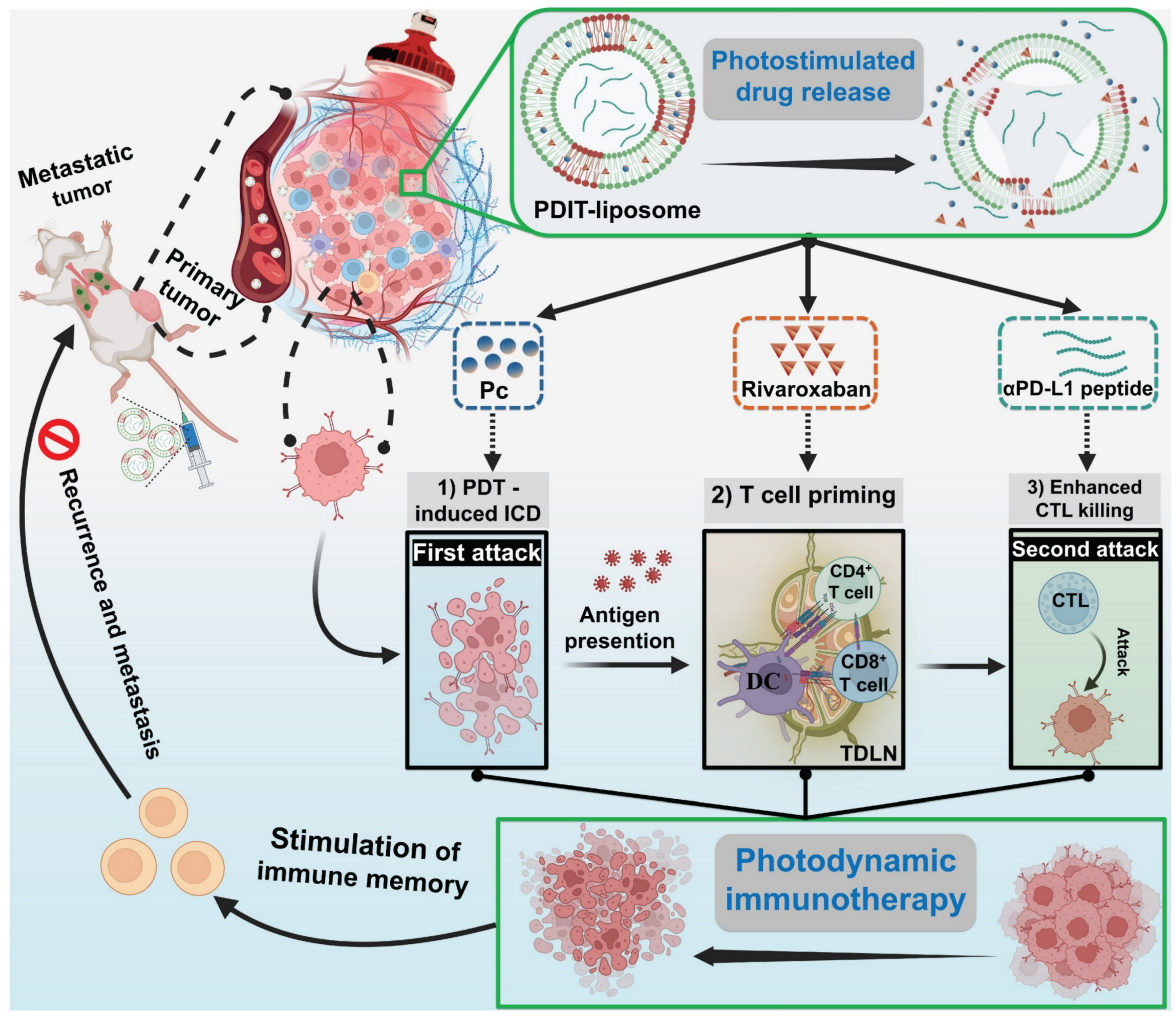
Schematic illustration of the mechanism of photo-controlled PDIT effect. (A) PDIT-liposome is fabricated using the ROS-sensitive DOPC as the lipid scaffold, encapsulating Pc, rivaroxaban, and αPD-L1. (B) Upon passive targeting and accumulation in tumor tissues, Pc-mediated PDT generates ROS to disrupt the liposomal scaffold releasing the three components: First, Pc-mediated PDT triggers the first attack by inducing ICD of tumor cells exposing TAAs and DAMPs; Second, rivaroxaban modulates coagulation-immune crosstalk and promotes T-cell priming in both TDLNs and tumor tissues; Finally, αPD-L1 blocks immune checkpoint reducing T-cell exhaustion and potentiating CTL's second attack. The synergism of three components amplifies PDT-induced antitumor immunity, achieving enhanced therapeutic efficacy.

The rationale underlying the synergism between anticoagulation therapy and PD1/PD-L1 inhibition lies in their targeting of distinct stages of tumor immune escape. Thus, combining both with PDT is hypothesized to achieve a more comprehensive inhibition of immune escape compared to single-component combinations (PDT + checkpoint inhibition or PDT + anticoagulation therapy). To this end, we designed a liposomal nanomedicine for photodynamic immunotherapy (PDIT-liposome) encapsulating three key components: a phthalocyanine-based photosensitizer (Pc) for PDT, a FXa inhibitor (rivaroxaban) to modulate coagulation-driven immunosuppression, and a peptide inhibitor of PD-L1 (αPD-L1) to reverse T-cell exhaustion (Scheme 1). The liposome scaffold was constructed with ROS-sensitive phospholipid, enabling controlled drug release in response to the light-triggered PDT 29. Mechanistically, PDIT-liposome operates through a sequential process: Upon light exposure, Pc generates ROS oxidizing DOPC and disrupting the liposomal structure to release the encapsulated therapeutics. First, Pc-mediated phototoxicity directly destructs subcellular organelles inducing ICD, TAAs exposure and DAMPs release. Consequently, released rivaroxaban inhibits myeloid-derived FXa, promoting antigen presentation, DC maturation, and T-cell priming, which also facilitates infiltration of immune cells in the TME. Finally, αPD-L1 blocks PD-1/PD-L1 signaling and reprograms exhausted CTLs, enhancing elimination of tumor cells. By integrating direct tumor cell destruction (PDT), modulation of coagulation-driven immune suppression (rivaroxaban), and reinvigoration of exhausted CTLs (αPD-L1), this PDIT-liposome platform demonstrates potent and durable antitumor responses against both localized and disseminated malignancies. We systematically characterized the formulation's physicochemical properties, drug release profiles, and *in vivo* tumor targeting, followed by detailed efficacy assessments in murine tumor models to elucidate the immunomodulatory mechanisms underlying this tripartite synergy. This work exemplifies an advanced delivery science approach to potentiate and sustain photodynamic immunotherapy, providing insights for translational development of nanomedicine-enabled combinational cancer therapies.

## Materials and Methods

### Materials

Mono-substituted β-carboxy phthalocyanine zinc (Pc) was synthesized as previously reported 30. Cholesterol, DPPC, DOPC, and rivaroxaban (Shanghai Yuanye Bio-Technology), Tween-80 (Shanghai Aladdin Biochemical Technology), Chloroform and methanol (Sinopharm Chemical Reagent), and side chain-protected αPD-L1 peptide on Wang resin (Sangon Biotech) were used as supplied by the manufacturers without additional purification. All animal experiments complied with the National Research Council's Guide for the Care and Use of Laboratory Animals and were approved by the Animal Ethics Committee of the College of Biological Science and Engineering, Fuzhou University (2021-SG-072) and carried out in strict accordance with the guidelines. Additional material information is available in the Supplementary material.

### Synthesis of PDIT-liposome

The film hydration method was employed to fabricate PDIT-liposomes 31. Cholesterol, DPPC, DOPC, Pc, rivaroxaban, αPD-L1 peptide, and Tween-80 (molar ratio% = 36.27: 49.46: 4.4: 0.21: 3.79: 0.09: 5.78) were precisely weighed and dissolved in 6 mL of chloroform: methanol (v: v = 1:1) mixture. Following sonication to ensure complete dissolution, the organic solvent was removed under reduced pressure at room temperature to form a homogeneous lipid film. Vacuum treatment was applied for 4 h at room temperature to ensure complete elimination of residual organic solvents. Subsequently, 6 mL of PBS (pH 7.4) was added to the dried lipid film, and hydration was performed at 40 °C with orbital shaking at 180 rpm for 8 h. The resulting liposome suspension was sonicated at 4 °C for 10 min to achieve size homogenization. Aggregates and large particles were removed by centrifugation at 4,000 rpm for 5 min. The supernatant was filtered through a 0.22 μm filter membrane and subsequently extruded using a liposome extruder (Avanti mini-extruder 610000, Avanti Polar Lipid, USA) to refine particle size distribution. Finally, liposomes were dialyzed against PBS for 24 h at 4 °C to remove unencapsulated drug and organic solvent residues. For control liposome preparations, the identical film hydration protocol was employed. Blank control liposome (no active components), P-liposome (Pc alone), RD-liposome (rivaroxaban + αPD-L1 peptide), PD-liposome (Pc + αPD-L1 peptide), and PR-liposome (Pc + rivaroxaban) were prepared using equivalent molar ratios of respective components while maintaining the core lipid scaffold composition.

### Physicochemical characterization and stability of PDIT-liposome

The optical characterization of PDIT-liposome included UV-vis absorption and fluorescence spectra measurements using multifunctional microplate reader (BioTek Instruments, Winooski), complemented by fluorescence imaging by a molecular tomography 2500TM LX instrument (PerkinElmer, Waltham, MA). Particle size distribution and polydispersity index (PDI) were determined by dynamic light scattering (DLS, Zetasizer Nano ZS, Malvern Panalytical). Liposomes' morphology was characterized by scanning electron microscopy (SEM, Verios G4, Thermo Fisher Scientific), transmission electron microscopy (TEM, Hitachi HT7700) and atomic force microscopy (AFM, Agilent 5500). To stability of liposomes stored at 4 °C, RT, and 37 °C for seven days was assessed by monitoring of size and PDI daily. Batch-to-batch reproducibility was verified through triplicate preparations analyzed under identical conditions.

### Encapsulation efficiency (EE%), loading efficiency (LE%) and light-triggered drug release

The EE% of Pc, rivaroxaban and αPD-L1 in liposomes was measured using indirect centrifugation method. Briefly, liposomes were centrifuged at 14,000 rpm for 30 min, and supernatants were analyzed via high-performance liquid chromatography (HPLC, Sinochrom ODS-BP P230P, equipped with a C18 column, 1 mL/min flow rate) with component-specific conditions: Pc was detected at 670 nm using a 30-min H₂O/DMF gradient (50-100%, 0.1% TFA); rivaroxaban was detected at 270 nm with a 30-min H₂O/DMF gradient (10-90%, 0.1% TFA); and αPD-L1 was detected at 280 nm via a 22-min H₂O/ACN gradient (10-75%, 0.1% TFA). EE% was calculated according to the following equation: EE% = (S_total_ - S_free_)/S_total_×100%, where S_total_ and S_free_ are the peak area of the total feed and supernatant of each component, respectively. LE% was calculated according to the following equation: LE% = (S_compo_ / (S_lipo_ + S_compo_) × 100%, where S_compo_ and S_lipo_ are the weight of each component encapsulated in the liposome and the weight of the carrier, respectively. For light-triggered drug release, PDIT-liposome was irradiated with a 680nm LED light source (40.5 J/cm²). ROS and singlet-state oxygen (^1^O_2_) generation were assessed using DCFH-DA and DPBF as fluorescent probes, respectively, as described in our previous study 32. Post-irradiation changes were evaluated through: (1) DLS to monitor particle size distribution changes, (2) TEM imaging to assess morphological alterations, and (3) enzymatic kinetics of FXa to verify rivaroxaban's inhibitory potency. More details were shown in the Supplementary material. Time-dependent release kinetics were studied in dark conditions and under light irradiation (680 nm, 45 mW/cm^2^) at identical time points. Released components were quantified by HPLC using the aforementioned conditions, with release rate calculated according to the following equation: S_t_/S_0_ × 100%, where S_0_ is the peak area of Pc, rivaroxaban or αPD-L1 peptide contained in liposomes, S_t_ is the peak area of Pc, rivaroxaban or αPD-L1 peptide contained in dialysate at each time point. All release experiments were conducted in triplicate using dialysis membranes (MWCO 1.5 kDa) with continuous agitation (50 rpm) at 37 °C in PBS containing 0.1% Tween-80.

### *In vitro* ICD assessment

Human colorectal cancer HCT-116 cells (Shanghai Institute of Cell Biology) and CT-26 murine colorectal cancer cells (Shanghai Institute of Cell Biology) were seeded and incubated with various liposomes (control, P-, RD-, or PDIT-liposome) at a concentration of 2 mg/mL for 6 h. Following incubation, cells were exposed to light irradiation (680 nm, 1.5 J/cm²) and were further incubated for an additional 4 h. Cell culture supernatants were collected from each group to quantify extracellular released adenosine triphosphate (ATP) using an ATP assay kit. To evaluate surface-exposed calreticulin (ecto-CRT), cells were washed and incubated with an ecto-CRT-specific FITC-labeled peptide probe (CRTpep-FITC) for 4 h 33, followed by nuclear staining with Hoechst 33342 for 0.5 h. To evaluate the release of HMGB1, cells after treatments were washed twice with cold PBS and fixed with 4% paraformaldehyde for 20 min at room temperature. Next cells were incubated with primary antibodies against HMGB1 at 4 °C overnight, followed by thorough washing and incubation with Alexa 594-labeled secondary antibody at 37 °C for 1 h. Nuclei were stained using Hoechst 33342 for 0.5 h. All fluorescence imaging was performed using high-content analysis system (Operetta CLS, PerkinElmer, Waltham, MA). Detailed protocols for cell culture and cytotoxicity assays are provided in the Supplementary Material.

### Animal model establishment, imaging, and therapeutic evaluation

Male BALB/c mice (18-22 g, 20 weeks) were subcutaneously implanted with the mouse colorectal cancer CT-26 cells (5×10⁷ cells/mL, 100 μL) in the right dorsal flank. Tumor-bearing mice were employed upon tumor volume reaching 100-200 mm³. For fluorescence imaging, tumor-bearing mice administrated with PDIT-liposome (2 mg/kg, i.v.) underwent *in vivo* imaging at 0, 3, 6, 9, 12, 24, 36, 48, and 72 h post-injection using a Fluorescence Molecular Tomography system (PerkinElmer; excitation: 680 nm, emission: 690 nm), with a 3D reconstruction and quantification module via TrueQuant v3.0 software (PerkinElmer, Waltham, MA). Tissue distribution of PDIT-liposome was analyzed 9 h post-injection by *ex vivo* imaging of dissected organs (liver, lung, spleen, kidney, heart, tumour, and brain) according to our standard protocol 34. For therapy, tumour-bearing mice were randomized into 5 groups (n = 6) and treated with various liposomes (control, P-, PR-, PD-, and PDIT-liposome) at an identical concentration of 2 mg/kg, and received tumor-localized NIR irradiation (680 nm, 40.5 J/cm²) at 9 h post-injection. Tumor volumes (0.5 × Length × Width^2^) and body weights were monitored for 8 days. On day 8th, mice were sacrificed, and tumor tissues were resected for quantitation and histopathological analysis.

### Histopathological analysis

Histopathological sections were prepared by Wuhan Servicebio Technology Co., Ltd. Tumor tissues were stained with Hematoxylin and Eosin staining (H&E), TUNEL (apoptosis), and Ki67 (proliferation), CCL5 and CCR5 (chemokines), respectively. TUNEL-, Ki67- positive, CCL4^+^ and CCR5^+^ cells were quantified using Image J (National Institutes of Health, USA). For immunohistochemistry (IHC) analysis, tumor and TDLNs sections were stained against primary antibodies of CD8, CD4, CD107a and CD11c. Positive cells were quantified using ImageJ software 35. The percentage of positive cells was graded into four classes: 0 as < 5%; 1 as 6%-25%; 2 as 26%-50%; 3 as 51%-75% and 4 as > 75%. Staining intensity was assessed by 4 degrees: 0, negative; 1, weak; 2, moderate; and 3, strong. Staining results were evaluated semi-quantitatively by calculating the IHC score. IHC score can be calculated using the following formula: IHC score = cell staining intensity score × percentage of positive cells score. For immunofluorescent (IF) analysis, tumor and TDLNs sections were double-stained with CD8 and CD11c. The CD8^+^CD11c^+^ positive DCs were quantified using Image J software.

### Lung metastatic model

The lung metastatic model was established based on CT-26 cells stably transfected with mCherry genes (CT-26-mCherry) reported in our previous study 36. Male Balb/c mice (~20 g) were divided into 5 groups (n = 6 mice per group) and subcutaneously implanted with CT-26-mCherry cells (5×10^7^ cells/mL, 100 μL). Tumor-bearing mice were treated with 2 mg/kg various liposomes (control, P-, PR-, PD-, and PDIT-liposome) and illuminated with an NIR light source (680 nm, 40.5 J/cm^2^) at 9 h post-injection, respectively. On day 4 post-treatment, 200 μL of CT-26-mCherry cell suspension (1×10^6^ cells) was injected via the tail vein. On day 15, mice were euthanized and sacrificed, and the lung tissues were harvested and weighed immediately. The mCherry fluorescence in the lung tissues was imaged using an Amersham Imager 600 *in vivo* fluorescence imager (GE Healthcare Bio-Sciences AB) with 590 nm laser diode excitation. Fluorescence signals were quantified by collecting fluorescence signals within a 20 × 20 mm^2^ area. The metastatic nodules on the lung surface were recorded. The lung tissue was sent for histopathological analysis.

### Statistical analysis

All data are presented as mean ± standard deviation (SD) based on 3-8 independent replicates. The statistical significance was analyzed using 1-way ANOVA with Dunnett multiple comparison test or 2-way ANOVA with Sidak multiple comparisons test. A *P* value < 0.05 was considered statistically significant.

## Results

### Synthesis and physicochemical characterization of multi-therapeutic PDIT-liposome

**Scheme 2:**
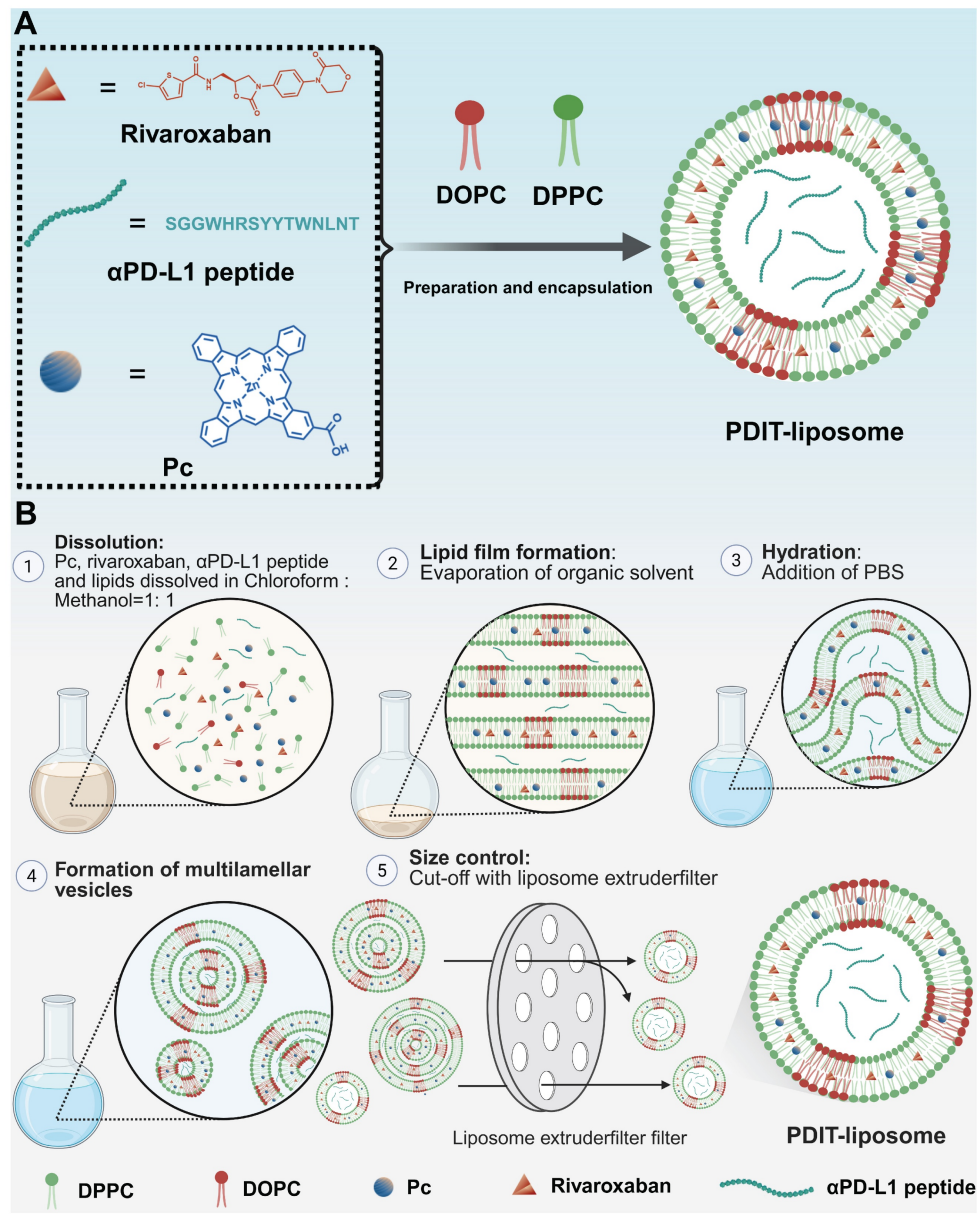
** Synthetic procedure of PDIT-liposome.** (A) Schematic illustration of the construction of PDIT-liposome. PDIT-liposome is fabricated using the ROS-sensitive DOPC as the lipid scaffold, encapsulating Pc, rivaroxaban, and αPD-L1. (B) The molar ratio% of each component is DPPC: DOPC: Cholesterol: Pc: rivaroxaban: αPD-L1: Tween-80 = 49.46: 4.4: 36.27: 0.21: 3.79: 0.09: 5.78.

**Figure 1:**
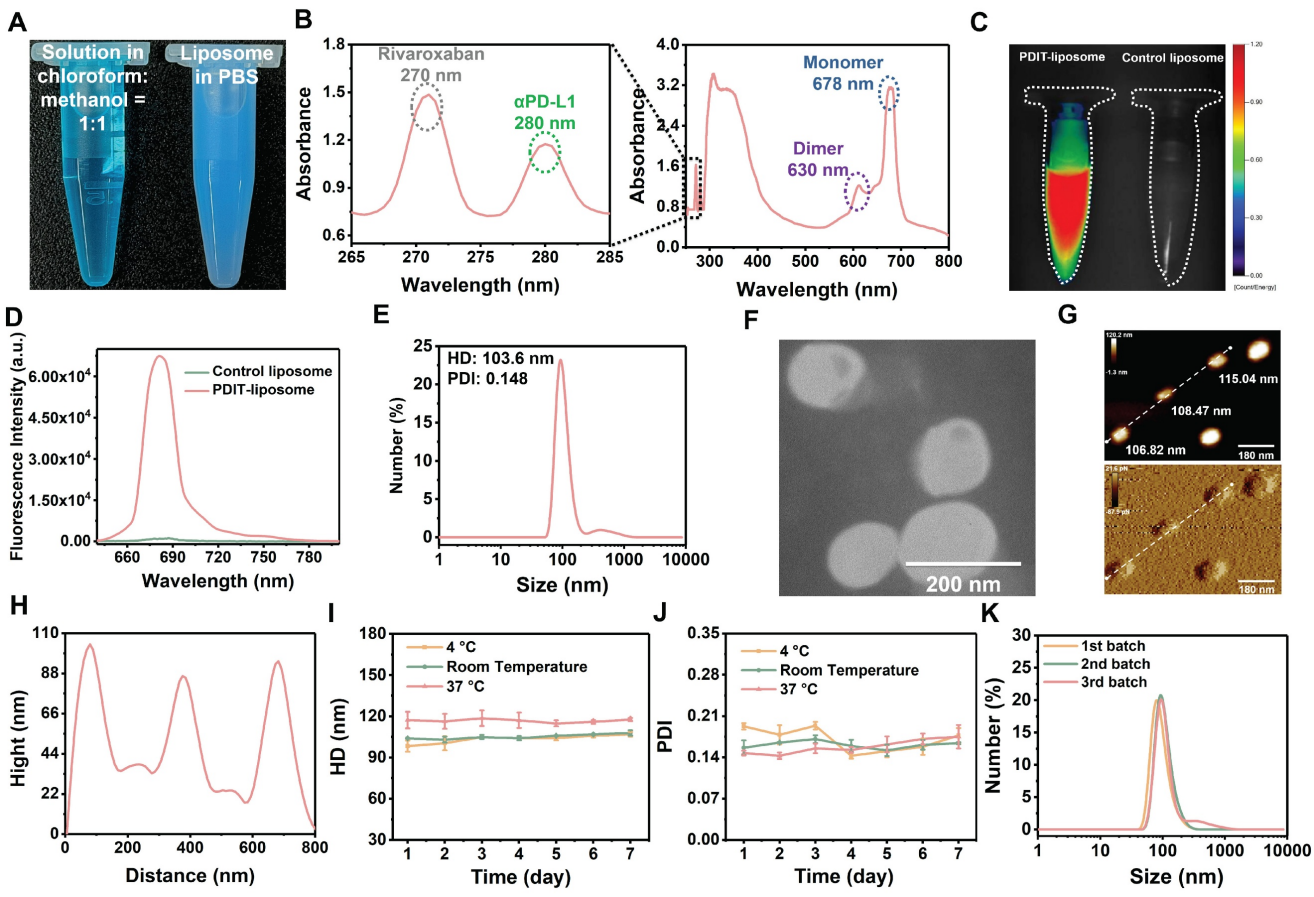
** Characterization of PDIT-liposome.** (A) Visual comparison of the chloroform: methanol =1:1 solution containing three components and phospholipids (left) and PDIT-liposome dispersed in PBS (right); (B) UV-vis absorption spectrum of PDIT-liposome, showing characteristic absorbance of rivaroxaban (270 nm), αPD-L1 (280 nm), and Pc (678 nm); (C) Fluorescence imaging (ex630 nm) and (D) Fluorescence emission spectrum of PDIT-liposome (ex610 nm), hydrodynamic diameter (E) and TEM image (F) of PDIT-liposome. (G-H) AFM topography (G) and height profiles (along the white line in G) of PDIT-liposome (H); (I-K) Stability evaluation of PDIT-liposome by daily average HD (I) and PDI (J) of PDIT-liposome during storage at 4 °C, room temperature, and 37 °C; (K) Batch-to-batch reproducibility analysis: size distribution of PDIT-liposome across different batches measured by DLS.

The peptide inhibitor of PD-L1, αPD-L1, was synthesized via solid-phase peptide synthesis method and structurally characterized by mass spectrometry (Figure S1). Surface plasmon resonance (SPR) analysis confirmed αPD-L1's equilibrium dissociation constant (*K_D_*) for binding to PD-L1 was 10.62 μM (Figure S2). A hydrophobic asymmetric zinc phthalocyanine (Pc) reported in our previous study was chosen as the photosensitizer 37. PDIT-liposomes were synthesized via the thin-film hydration method, incorporating Pc, αPD-L1, and rivaroxaban into ROS-sensitive DOPC-doped liposome scaffolds (Scheme 2). For comparison, liposomes containing sole Pc, rivaroxaban/αPD-L1, Pc/αPD-L1 or Pc/rivaroxaban were synthesized and termed as P-liposome, RD-liposome, PD-liposome and PR-liposome, respectively. Before liposome formation, three components showed a blue transparent solution in the chloroform : methanol = 1:1 solution, while the aqueous dispersion of PDIT-liposome appeared blue, opaque, and turbid (Figure 1A). UV-Vis absorption spectroscopy confirmed the successful encapsulation of three components by showing the characteristic Q-band of Pc (600-800 nm), the characteristic absorption of rivaroxaban (270 nm), and tryptophan in the αPD-L1 (280 nm) (Figure 1B). Notably, the strong Q-band at 678 nm (monomeric Pc) and reduced shoulder at 630 nm (aggregated Pc) indicated that Pc were predominantly in the monomeric form within PDIT-liposome, which was further validated by the strong fluorescence emission in aqueous solution (Figure 1C-D). DLS analysis revealed the hydrodynamic diameter (HD) of 103.6 nm and a PDI of 0.148 for PDIT-liposomes in aqueous solution (Figure 1E). SEM (Figure S3), TEM (Figure 1F), and AFM (Figure 1G-H) imaging confirmed the spherical morphology of PDIT-liposomes with sizes of 101.55 nm, 130.17 nm, and 110.11 nm, respectively, which largely consist with the HD in aqueous solution determined by DLS (Table S1). Storage stability assays demonstrated that the HDs of PDIT-liposome in PBS remained stable over 7 days at 4 °C, room temperature, and 37 °C, with the PDI values fluctuating minimally in the range of 0.143-0.193 (Figure 1I-J). Additionally, the batch-to-batch reproducibility was confirmed by consistent HD and PDI across independent preparations (Figure 1K, Table S2).

### Phototriggered PDIT-liposome disruption and controlled release

**Figure 2:**
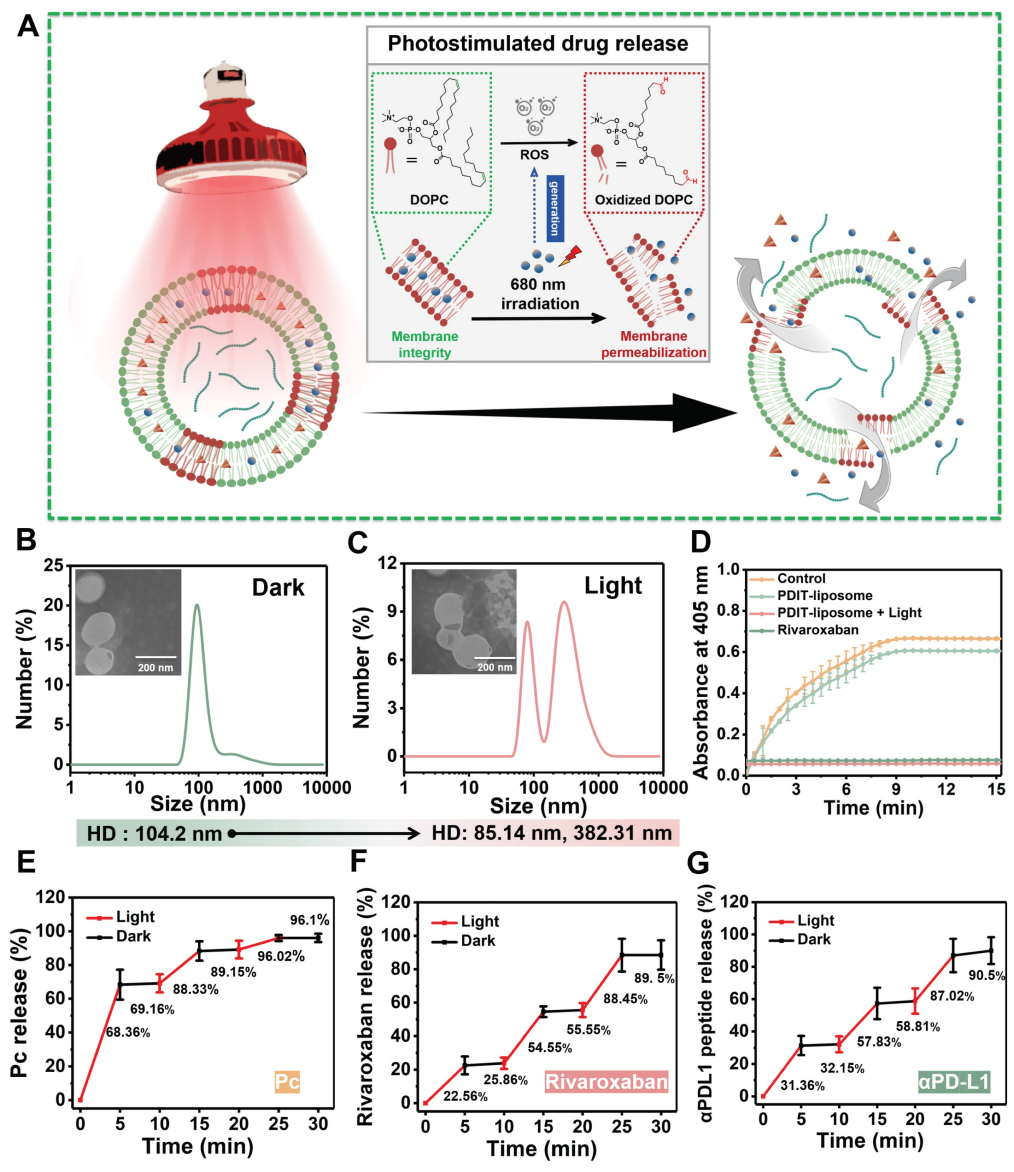
** Photostimulated drug release of PDIT-liposome.** (A) Schematic illustration of the photostimulated drug release process. (B-C) TEM images (insets) and HD analysis of PDIT-liposome before (B) and after (C) light irradiation (680 nm, 40.5 J/cm^2^); (D) Enzymatic activity of factor Xa in the presence of rivaroxaban, PDIT-liposome with or without light illumination (680 nm, 40.5 J/cm^2^). Enzymatic activity of FXa was quantified by monitoring OD405 changes of S-2765. (E-G) Time-dependent release profiles of Pc (E), rivaroxaban (F), and αPD-L1 (G) in dark conditions (black) and after light irradiation (red, 680 nm, 45 mW/cm^2^) at identical time points. Data are presented as Mean ± SD (n = 3).

The EE% of Pc, rivaroxaban, and αPD-L1 in PDIT-liposomes were quantified using HPLC chromatography. The average EE% was 91.8% for Pc (Figure S4A), 93.67% for rivaroxaban (Figure S4B), and 95.55% for αPD-L1 (Figure S4C). Notably, batch-to-batch consistency was confirmed with no significant differences in EE% and LE% across independent preparations (Table S2, S3). As the doping of ROS-sensitive DOPC into the liposomal scaffold enables the photo-triggered controlled drug release (Figure 2A), we assessed ROS and ^1^O_2_ generation by PDIT-liposome using DFCH-DA and DPBF as probes, respectively (Figure S5). DLS analysis revealed that the HD of non-irradiated PDIT-liposome remained stable at 104.2 nm, in sharp contrast to the split fragments of 85.14 and 382.31 nm after irradiation (680 nm, 40.5 J/cm^2^) (Figure 2B-C). Accompanied by a significant increase in PDI (0.431), the disrupted integrity of liposomal scaffold was confirmed. Consistently, TEM also showed liposomal membrane rupture in irradiated PDIT-liposomes (Figure 2B-C).

The release of rivaroxaban was also certified by assessing the inhibition of factor Xa's enzymatic activity by PDIT-liposome with or without irradiation. In contrast to the non-inhibition by PDIT-liposome without illumination, Irradiated PDIT-liposomes effectively inhibited FXa activity (Figure 2D). In addition, HPLC-based quantification of drug release kinetics under light and dark conditions at identical time points demonstrated that drug release is specifically triggered by ROS generated through Pc-mediated photochemistry, not passive diffusion or time-dependent degradation. A light dose-dependent release profile: 96.1% ± 2.5% of Pc, 89.5 ± 8.81 of rivaroxaban, and 90.5 ± 8.3 % of αPD-L1 were released within total 40.5 J/cm^2^ of irradiation (Figure 2E-G, S6).

### *In vitro* photodynamic cytotoxicity and ICD

**Figure 3:**
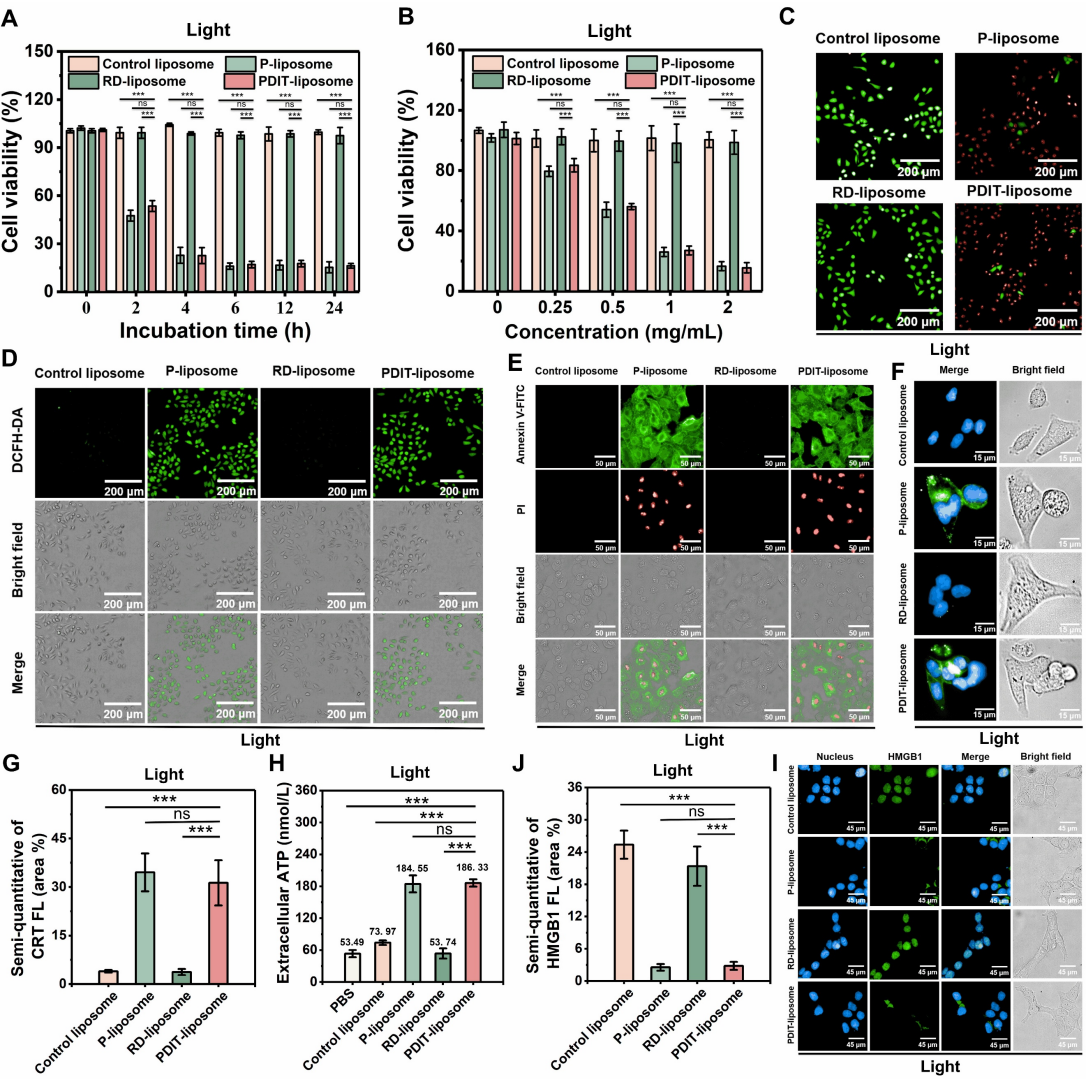
** Antitumor efficacy of PDIT-liposome *in vitro*.** (A) Incubation time-dependent (2 mg/mL) and (B) drug dose-dependent (1.5 J/cm^2^) cytotoxicity of control liposome, P-liposome, RD-liposome, and PDIT-liposome against HCT-116 cells. Photocytotoxicity was triggered by irradiation with a 680 nm LED light source. (C) Representative live/dead staining images of HCT-116 cells treated with various liposomes after irradiation (680 nm, 1.5 J/cm^2^). Live and dead cells were fluorescently imaged by Calcein-AM (green, ex480/em500) and PI (red, ex490/em635), respectively. (D) Intracellular ROS generation by PDIT-liposome after irradiation. ROS was imaged with DCFH-DA as the fluorescent probe. (E) Fluorescent imaging of apoptotic and necroptotic HCT-116 cells stained with Annexin V-FITC (green, ex494/em518) and PI (red, ex490/em635) after irradiation (680 nm, 1.5 J/cm^2^). (F) Fluorescent imaging of CRT exposure on the surface of HCT-116 cells stained with Hoechst 33342 (blue, ex350/em460) and ecto-CRT targeted imaging probe, CREpep-FITC (green, ex485/em538). Cells were treated with various liposomes (2 mg/mL) for 6 h, followed by irradiation (680nm, 1.5 J/cm^2^) before imaging. (G) Corresponding fluorescence intensity was semi-quantitatively calculated using ImageJ software. (H) Extracellular secretion of ATP in HCT-116 cells after irradiation (680 nm, 1.5 J/cm^2^). (I) Fluorescent imaging of HMGB1 release from HCT-116 cells stained with Hoechst 33342 (blue, ex350/em460), primary antibodies against HMGB1 and Alexa Fluor 594-conjugated secondary antibody (green, ex594/em617). Cells were treated with various liposomes (2 mg/mL) for 6 h, followed by irradiation (680 nm, 1.5 J/cm^2^) before imaging. (J) Corresponding fluorescence intensity was semi-quantitatively calculated using ImageJ software. Data are presented as Mean ± SD (n = 6, **P < 0.01, ***P < 0.001).

To investigate the *in vitro* antitumor PDT efficacy of PDIT-liposome, we evaluated the cytotoxicity of PDIT-liposome against human colorectal cancer cell line (HCT-116) and murine colorectal cancer cells (CT-26) with or without light illumination, using an empty liposome (control liposome), P-liposome, and RD-liposome as controls. In the absence of light irradiation, all liposome groups showed no measurable cytotoxicity (cell viability > 95%) (Figure S7A, S7B). Under light irradiation (680 nm, 1.5 J/cm^2^), The IC_50_ values for P-liposome and PDIT-liposome reached 0.57 mg/mL and 0.61 mg/mL, respectively, both P-liposome and PDIT-liposome displayed incubation time-dependent (Figure 3A) and concentration-dependent (Figure 3B) phototoxicity, reaching saturation at 6 h post incubation with a dose of 2 mg/mL and light dose of 1.5 J/cm^2^ (680 nm). In addition, both PR-liposome and PD-liposome exhibited similar dark toxicity and phototoxicity to P-liposome and PDIT-liposome (Figure S8). This result consists with the known non-cytotoxicity of rivaroxaban and αPD-L1. Additionally, both of them have no potentiation effect on the phototoxicity of Pc. Phototoxicity and dark cytotoxicity of liposomes were further confirmed based on the live/dead cell staining (Figure 3C, S9), which demonstrates significant cell death in the P liposome and PDIT-liposome groups under light irradiation. Intracellular ROS generation in CT-26 cells by P-liposome and PDIT-liposome was also verified using DCFH-DA as a ROS probe (Figure 3D, S10). To characterize the mode of cell death, we used an AnnexinV-FITC (green)/Propidium Iodide (PI, red) apoptosis detection kit (Figure 3E, S11). In control liposome- and RD-liposome-treated cells, negligible green and red fluorescence was observed irrespective of light irradiation, which excludes the effect of light irradiation, dark toxicity of rivaroxaban and αPD-L1. In contrast, the groups treated with P-liposome and PDIT-liposome showed a significant increase in AnnexinV-FITC-positive cells, indicating the early apoptosis state. A small fraction of cells exhibited dual AnnexinV-FITC/PI staining, indicating necrosis. The ROS generated from PDT induced ICD characterized by the expression of DAMPs, including CRT exposure, extracellular ATP and HMGB1 release, which are critical for promoting DC maturation and antigen presentation. Fluorescence imaging analysis (Figure 3F-G, S12-13) showed minimal CRT exposure on the cell membrane in all groups without laser irradiation. In contrast, CRT exposure significantly increased upon laser irradiation, with the P-liposome and PDIT-liposome groups exhibiting the highest levels of CRT expression. Consistently, extracellular ATP secretion levels measured by ATP assay (Figure 3H, S14) were significantly elevated in both P-liposome and PDIT-liposome groups under laser treatment. IF analysis revealed significant green fluorescence detected in nucleus in all groups without laser irradiation. Following laser irradiation, green fluorescence was scarcely detectable in the nucleus of cells from the P-liposome and PDIT-liposome groups, indicating that nuclear HMGB1 had translocated to the cytoplasm and was subsequently released from tumor cells (Figure 3I-J, S15-16). Collectively, these results demonstrate that P-liposome and PDIT-liposome effectively induce apoptosis and necrosis through PDT-mediated phototoxicity primarily driven by phthalocyanine photosensitizer. This ICD subsequently facilitates DC maturation and activation of anti-tumor immune responses, thereby enhancing CTL-mediated tumor cell killing.

### Biocompatibility and safety profile of PDIT-liposome

**Figure 4:**
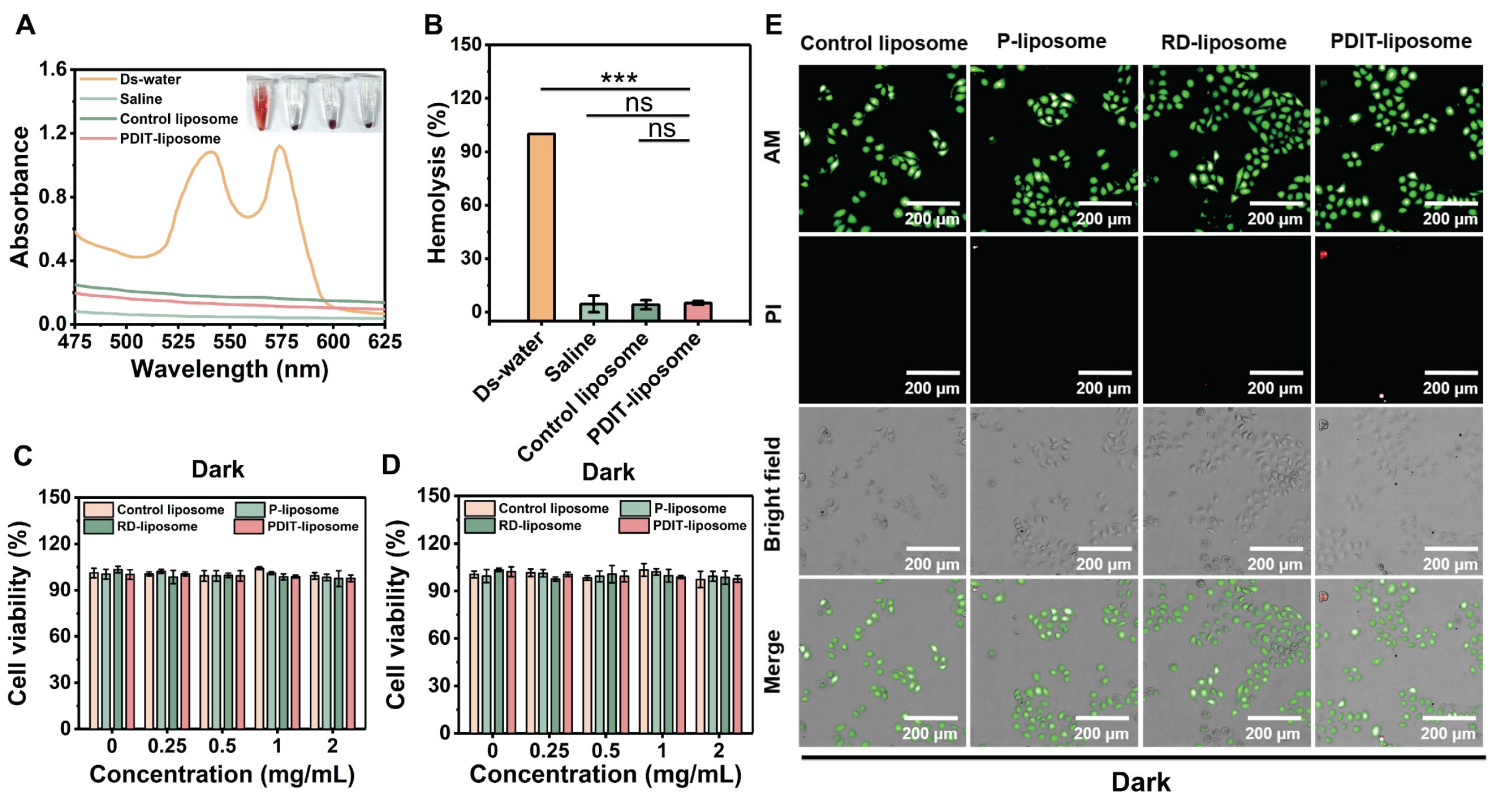
** Biosafety evaluation of PDIT-liposome.** (A) UV-vis absorption spectroscopy of supernatants from erythrocytes treated with ds-water, saline, control liposome, or PDIT-liposome, respectively. The inset shows representative visual images of the erythrocyte suspensions; (B) Quantitation of hemolytic rates in panel A by setting A540 in ds-water as 100%. (C-D) Viability of EA.hy926 (C) and LO2 (D) cells treated with various liposomes determined by the CCK8 assay. (E) Representative live/dead staining images of LO2 cells treated with various liposomes. Live and dead cells were fluorescently imaged by Calcein-AM (green, ex480/em500) and PI (red, ex490/em635), respectively. Data are presented as Mean ± SD (n = 6, **P < 0.01, ***P < 0.001 *vs* ds-water).

To evaluate the biosafety of our PDIT-liposome, we performed hemolysis assays and cytotoxicity tests on normal cell lines. First, hemolysis assays demonstrated that neither control liposomes nor PDIT-liposome induced significant hemolysis (hemolysis rate < 5%) (Figure 4A-B). Similarly, no obvious cytotoxicity was observed in two normal cell lines, human endothelial cell line (EA.hy 926) and human normal hepatocytes (LO2) after incubation with control liposomes or PDIT-liposome for 24 h, respectively (cell viability > 95%) (Figure 4C-D). The viability of LO2 cells was also confirmed by live-dead fluorescence staining (Figure 4E). Furthermore, blood biochemical analysis results showed no statistically significant differences in any biochemical parameters between the PDIT liposome group and the saline group (Table S4). Evaluation of activated partial thromboplastin time (APTT) or prothrombin time (PT) indicated that PDIT liposome group showed no statistically significant prolongation of APTT or PT compared with the saline group. All values remained within the normal physiological range (Table S5). These results confirm the low vascular toxicity and high biosafety of PDIT-liposome.

### *In vivo* tumor targeting, retention kinetics, and biodistribution of PDIT-liposome

**Figure 5:**
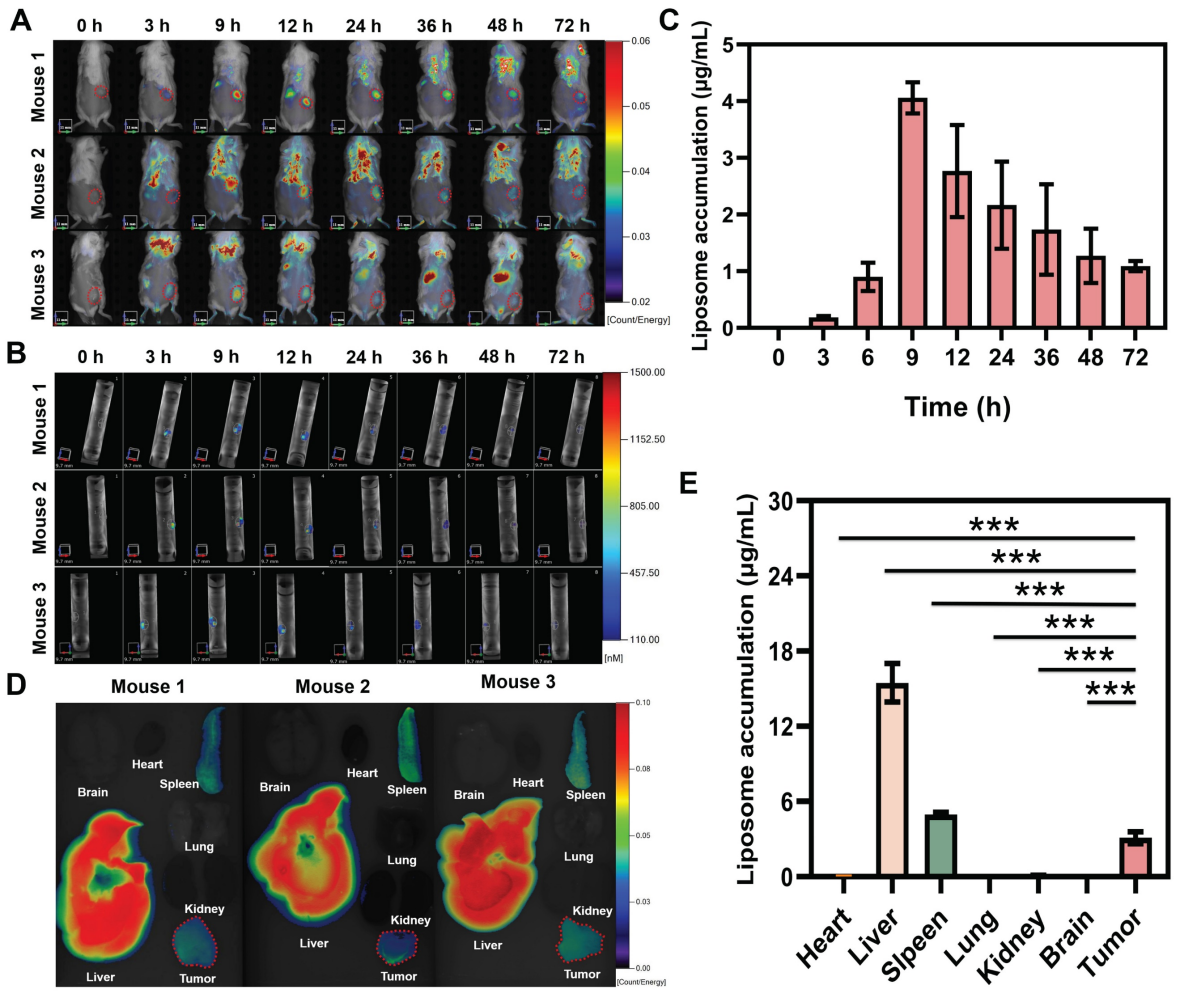
***In vivo* fluorescence imaging and biodistribution of PDIT-liposome in CT-26 tumor-grafted mice.** (A-B) Representative real-time 2D (A) and 3D (B) imaging of PDIT-liposome in CT-26-grafted mice. Tumor regions were highlighted with annuli. (C) Quantification of time-dependent PDIT-liposome accumulation at tumor sites (0-72 h post i.v. administration). (D-E) Representative images (D) and quantitative fluorescence intensity (E) of PDIT-liposome biodistribution in major organs (brain, heart, liver, spleen, lung, kidney) and tumor tissues collected at 9 h post i.v. administration (n = 6). Data are presented as Mean ± SD (n = 6, **P < 0.01, ***P < 0.001).

To investigate the tumor-targeting property of PDIT-liposome, we analyzed its *in vivo* retention kinetics at tumor sites and biodistribution in tumor-bearing mice. *In vivo* imaging revealed that PDIT-liposome selectively accumulated in tumor tissues, with fluorescence intensity peaking at 9 h post-administration (4.06 μg/mL) (Figure 5A-C). This time point was designated as the “peak accumulation time” for subsequent experiments. Biodistribution quantification at 9 h post-administration (Figure 5D-E) demonstrated that PDIT liposomes exhibited accumulation levels below 0.1 μg/mL in the brain, heart, and lungs, with no significant observed accumulation. In contrast, tumor tissues showed significantly higher PDIT-liposome accumulation in tumor tissue (3.11 μg/mL), consistent with the enhanced permeability and retention effect at tumor sites of other liposomes 38, 39. Notably. PDIT-liposome also showed elevated accumulation in the spleen and liver. Accumulation in the liver reached 15.4 μg/mL, indicating the liver as the main metabolic organ. Additionally, spleen accumulation reached 4.97 μg/mL, which may be attributed to the size and morphological characteristics of the liposomes: spherical liposomes with diameters of 100-200 nm can be mechanically retained through the slit-like structure of splenic sinusoidal capillaries or phagocytosed by macrophages, leading to their retention in the spleen. This pattern is consistent with the reported biodistribution of liposomal nanomedicines 40, 41.

### Enhanced antitumor efficacy of PDIT-Liposome in a murine subcutaneous tumor model

**Figure 6:**
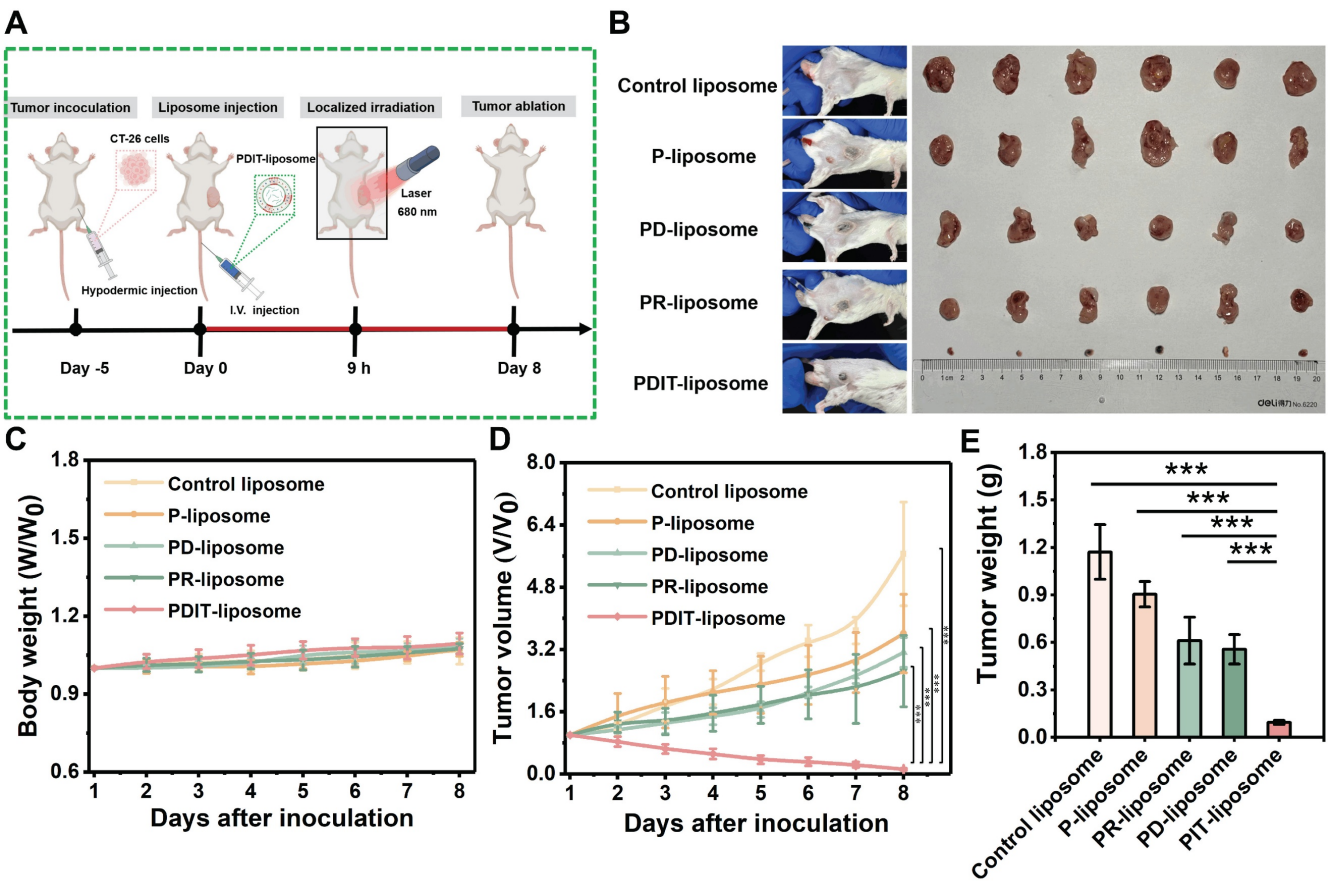
***In vivo* antitumor efficacy of PDIT-liposome in a subcutaneous tumor implantation model.** (A) Schematic diagram of the experimental workflow: CT-26-grafted mice were administrated (i.v.) with 2 mg/kg of various liposomes and illuminated (680 nm, 40.5 J/cm^2^) at 9 h post administration. Dynamic monitoring of body weight (C) and tumor volume (D) over 8 days post-illumination (n = 6); Representative images (B) and weights (E) of resected tumor tissues on day 8. (n = 6); Data are presented as Mean ± SD (n = 6, **P < 0.01, ***P < 0.001).

**Figure 7:**
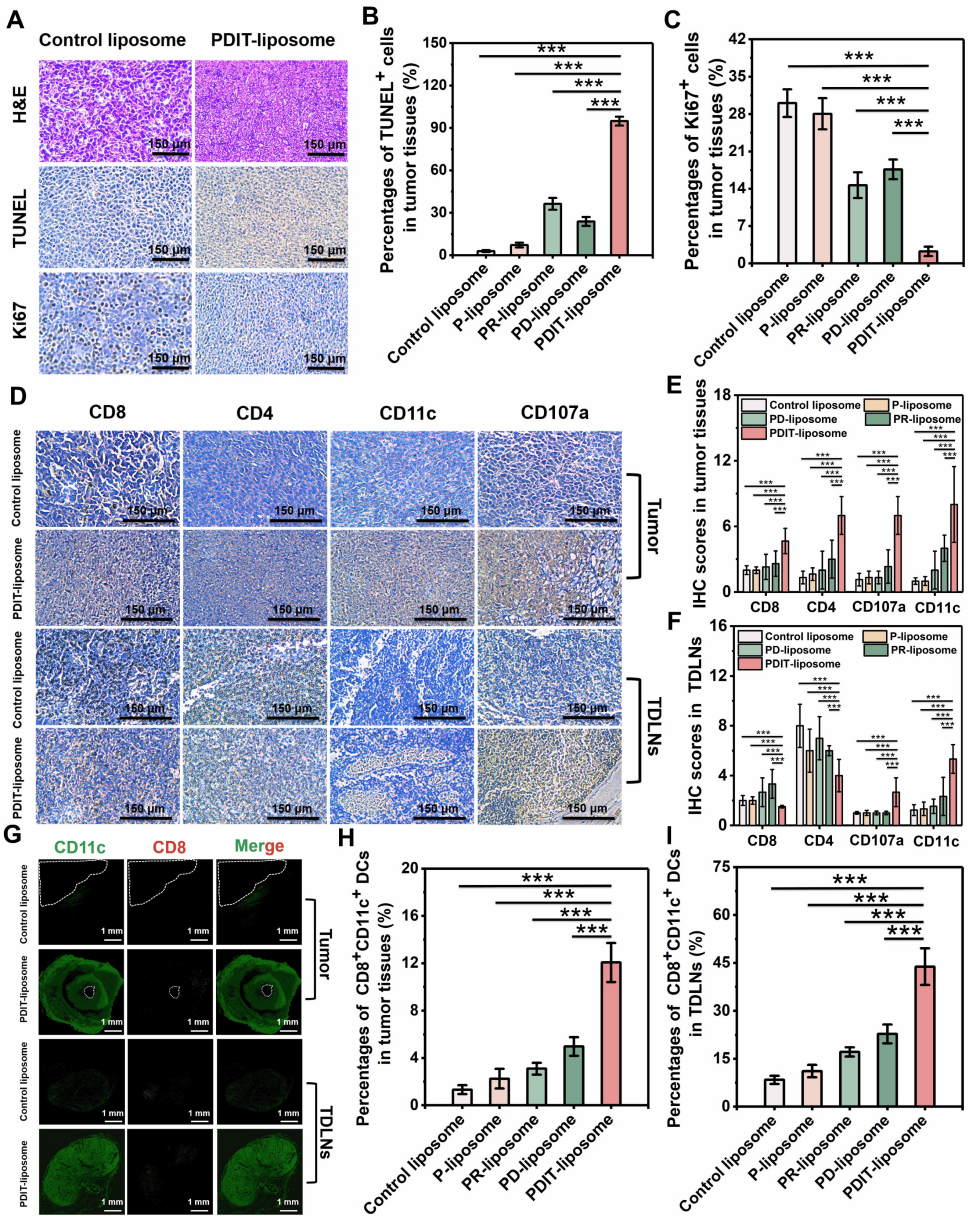
** Histopathological and immunohistochemical analysis of antitumor mechanisms of PDIT-liposome therapy.** (A) Representative histopathological sections of tumor tissues stained with H&E, TUNEL, Ki67, respectively. (B-C) Quantitation of TUNEL^+^ apoptotic cells (B) and Ki67^+^ proliferative cells (C) in tumor sections. (D) Hispopathological sections of tumor tissues and TDLNs stained with CD8, CD4, CD107a, and CD11c, respectively. (E-F) IHC scores for quantifying the density of CD8^+^, CD4^+^, CD107a^+^, CD11c^+^ cells in tumour tissues (E) and TDLNs (F). (G) Immunofluorescent analysis of tumor and TDLNs double-stained with CD8 and CD11c. The white dashed coil represents the tumor region and the yellow merged fluorescence represents the CD8^+^CD11c^+^ positive DCs. (H-I) Quantitation of CD8^+^CD11c^+^ positive DCs in tumor sections (H) and TDLNs (I). Data are presented as Mean ± SD (n = 6, **P < 0.01, ***P < 0.001).

To further evaluate the synergistic therapeutic effects of PDIT-liposome on local tumor *in vivo*, we employed a subcutaneous CT-26 tumor-implantation mouse model. Control liposome, P-liposome, PD-liposome and PR-liposome were used as controls. The experimental workflow is illustrated in Figure 6A and detailed PDT treatment parameters are provided in Figure S17. First, body weight monitoring revealed that all treatment groups maintained increasing gradually throughout the observation period, indicating excellent tolerance to PDIT-liposome treatment (Figure 6C). According to the daily tumor volume measurements (Figure 6D), P-liposome only moderately suppressed tumor growth with no statistical significance compared to the saline group, likely due to the suboptimal light dosage (680 nm, 40.5 J/cm^2^). PR-liposome and PD-liposome exhibited comparable while higher efficacy compared to P-liposome. Notably, PDIT-liposome achieved the highest antitumor efficacy, with tumor tissues nearly completely eradicated on Day 8 (Figure 6B). Quantitative analysis of resected tumor weights confirmed antitumor rates of P-liposome, PD-liposome, PR-liposome, and PDIT-liposome were 21.83%, 46.78%, 51.08% and 91.7%, respectively, relative to the control liposome group (Figure 6E). Based on a Bliss model 42, we confirmed the synergistic effects of three components rather than the simple summation of their individual effects (Table S6). Histopathological analysis corroborated the same qualitative findings showing that PDIT-liposome-treated tumors displayed significantly expanded necrotic area compared to the control liposome group (Figure 7A, S18). TUNEL and Ki67 staining further confirmed that the PDIT-liposome group exhibited a 32.83-fold increase in apoptosis levels (Figure 7B), while proliferative tumor cells decreased 13.39-fold (Figure 7C) compared to the control liposome group. Collectively, these results underscore the synergistic effects of the three components in PDIT-liposome in the suppression of tumor proliferation in local tumor tissues.

### Immunomodulatory effects of PDIT-liposome: promotion of dendritic cell activation and T cell infiltration

To characterize the PDIT mechanisms of PDIT-liposome, we performed immunohistochemical and immunofluorescent analysis of tumor tissues from mice treated with control liposome, P-liposome, PD-liposome, PR-liposome and PDIT-liposome (Figure 7D-I, S19-20). Given the critical role of TDLN in antigen presentation and T cell priming 43, 44, immune cell distribution in TDLNs was also assessed. In tumor tissues, PDIT-liposome treatment markedly increased CD8^+^ and CD4^+^ T cells, CD11c^+^ dendritic cells, and CD107a^+^ activated CTLs. Compared with control liposomes, the IHC scores respectively increased by 2.3-, 5.4-, 8.0- and 5.4-fold, indicating enhanced antigen presentation, T-cell priming, and effector activation. Particularly, CD107a, a marker of CTL degranulation or cytotoxic marker, was significantly promoted in PDIT-liposome treated tumor tissues, indicating the enhanced level of activated CTLs ready for the release of cytotoxic molecules (perforin, granzyme, etc.). This effect can be attributed to the combination of rivaroxaban and αPD-L1 (Figure 7E). Notably, the relative decrease in CD4^+^ and CD8^+^ T-cell densities of the PDIT group in TDLNs was accompanied by strong upregulation of CCL4 in tumors and CCR5 on tumor-infiltrating CD8^+^ T cells, supporting a CCL4-CCR5-driven recruitment of T cells from lymph nodes into the tumor microenvironment (Figure S21). In addition, elevated level of DCs in TDLNs and tumor were observed in the PDIT-liposome group (Figure 7E-F), suggesting enhanced T cell priming and effector differentiation in the PDIT-liposome group, which can be attributed to the effect of rivaroxaban 27. The antitumor immune activation of CD8^+^ T cells depends on the cross-presentation of tumor antigens by CD8^+^ DCs in TDLNs and tumor 27, 45. Furthermore, immunofluorescence analysis (Figure 7G-I) respectively showed a 9.54- and 5.22-fold expansion of CD11c^+^ CD8^+^ dendritic cells in both tumors and TDLNs, consistent with enhanced cross-presentation of tumor antigens and more efficient priming of CD8^+^ T cells. Together, these data support a coherent mechanistic model in which PDIT-liposome simultaneously promotes CD8^+^ dendritic cell expansion, relieves TAM-mediated immunosuppression via rivaroxaban, enhances CCL4/CCR5-dependent T-cell trafficking, and sustains T-cell effector function through PD-L1 blockade, thereby achieving coordinated and durable antitumor immune activation.

### PDIT-elicited immune memory mediated suppression of tumor metastasis

**Figure 8:**
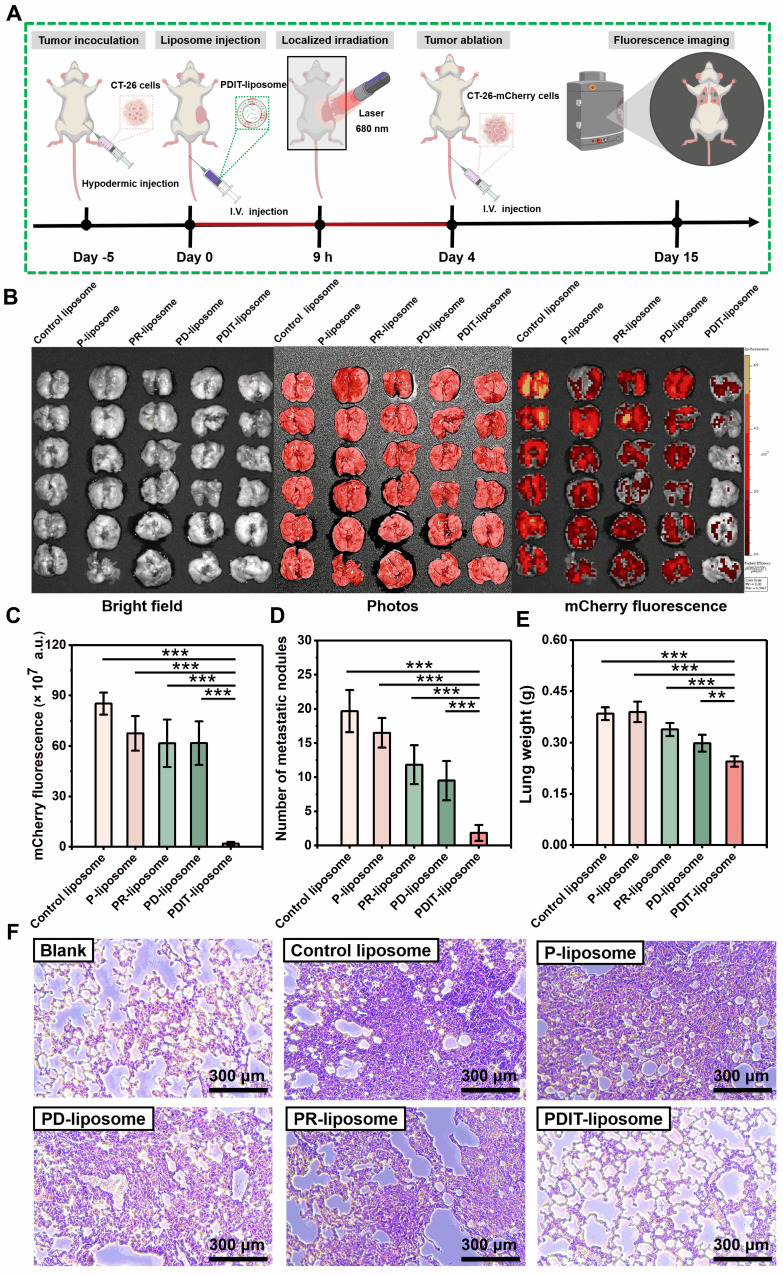
** PDIT-liposome-elicited immunological memory suppressed tumor metastasis.** (A) Schematic workflow of immunological memory, metastatic model establishment, and therapy process. (B) Images of lung tissues and fluorescence imaging of metastatic foci (mCherry+) in lung tissue. (C-E) Quantification of lung metastasis based on mCherry fluorescence intensity (C), nodule numbers on the lung surface (D) and lung weight increase (E). (F) Histopathological analysis of normal and metastatic lungs by H&E staining. Data are presented as Mean ± SD (n = 6, **P < 0.01, ***P < 0.001).

To investigate whether PDIT-liposome therapy elicits long-term immune memory against tumor recurrence and metastasis, we established a lung metastatic model (schematically elucidated in Figure 8A): Briefly, we first treated mice subcutaneously implanted with CT-26-mcherry cells using various liposomes to trigger the PDIT response as described in section 3.6. Four days after PDT therapy, 10^6^ CT-26-mCherry cells were intravenously injected to simulate tumor metastasis Lung metastasis was quantitatively assessed on Day 15 using three metrics: (1) mCherry fluorescence of metastatic CT-26-mCherry cells in the lungs, (2) numbers of surface metastatic nodules, and (3) metastasis-induced increase in lung weight.

Quantitative analysis of mCherry fluorescence revealed that PDIT-liposome treatment inhibited 97.8% of lung metastatic burden compared to the control liposome, markedly superior to other liposome groups (Figure 8B-C). This result was corroborated by metastatic nodule counting and lung weight increase, showing that PDIT-liposome group exhibited significantly fewer surface nodules and minimal lung weight increase compared to mice treated with other liposomes (Figure 8D-E). Quantitative inhibitory rates based on nodules counting of control liposome, P-liposome, PR-liposome, PD-liposome, and final PDIT-liposome were 16.1%, 39.83%, 51.69% and 90.68%, respectively.

Histopathological examination of lung tissues further confirmed our results (Figure 8F). In the control liposome group, extensive metastatic foci were observed throughout the lung parenchyma. Although other liposomes reduced lung metastasis to varying degrees, the PDIT-liposome group exhibited nearly complete suppression of metastatic lesions, with lung tissue morphology comparable to healthy mice. Thus, these results indicate that PDIT-liposome therapy elicits immune memory, which confers long-lasting protection against tumor recurrence and metastasis.

## Discussion

Despite its longer developmental history compared to emerging antitumor modalities like targeted therapy and immunotherapy, the progress of PDT is much slower 2. This lag can be attributed to multiple factors, including the technological limitations in the last century, the complexity of its mechanism, and limited therapeutic depth. Despite high effectiveness, PDT is limited by the lack of systemic persistence and treatment comprehensiveness because of the localized antitumor efficacy and its requirement for light illumination. In this context, phthalocyanine-based photosensitizers (PSs) offer distinct advantages over conventional porphyrin-based PSs for PDT applications, including higher ROS yield and therapeutic depth, lower skin phototoxicity 46, 47. The complementary nature PDT with targeted therapy or immunotherapy is thus logically compelling: while PDT excels in rapid, localized tumor ablation, it lacks systemic and long-lasting efficacy characteristic of targeted and immunotherapies. Recent interest has grown in photo-induced immune modulation, with studies showing that red or NIR light can modulate macrophages and lymphocytes' activities, thereby increasing immune responses to infections or injury 48. However, the underlying mechanism of such photo-induced immune modulation remains poorly understood. In contrast, PDT-mediated immune modulation in cancer treatment operates through well-characterized mechanisms. Beyond direct light-induced immune cell activation, PDT generates ROS via photochemical reactions, which not only includes antigen presentation through the induction of ICD of tumor cells, but also destroys immunosuppressive TME, like neovessels, cancer stem cells (CSC), and cancer-associated fibroblasts (CAF) 49, 50. Therefore, unlike immunosuppressive modalities like chemotherapy and radiotherapy, PDT revokes the immune responses for patients, which, however, are conventionally suppressed in TME, including the impaired T cell activation, CTL exhaustion, and DC dysfunction etc. Therefore, the synergism with immune checkpoint inhibitors has been broadly investigated to enhance the therapeutic efficacy of PDT.

Just as immunotherapy enhances the systemic efficacy, durability, and immune response of PDT, PDT also conversely potentiates immunotherapy, particularly immune checkpoint inhibitors, by transforming "cold tumors" into "hot tumors" through increased immune cell infiltration into tumor tissues 51. In the TME of cold tumors, insufficient T cells are present to effectively attack tumor cells, due to impaired antigen presentation or DC dysfunction. The induction of ferroptosis has been reported to improve the therapeutic outcome for cold tumors 52, because ferroptosis involves the Fenton reaction mediated by intracellular Fe^2+^ ions, which converts H_2_O_2_ into free radicals 53. These radicals oxidize polyunsaturated fatty acids in cell membranes, triggering the release of immunostimulatory signals such as HMGB1, CRT, ATP, and oxidized phospholipids (oxPLs). By enhancing tumor cell immunogenicity, ferroptosis facilitates the "cold-hot" transformation of tumor cells. Consequently, ferroptosis inducers (arachidonic acid and glutathione peroxidase 4 (GPX4) inhibitors) have demonstrated synergistic effects when combined with immune checkpoint inhibitors. However, ferroptosis exerts a dual role in tumor immunotherapy 54: CD8^+^ T cells are susceptible to ferroptosis due to GPX4 deficiency and CD36 overexpression, leading to the accumulation of lipid peroxides (LPO) and subsequent immunosuppression 55. Notably, PDT shares mechanistic similarity with the Fenton reaction in ferroptosis. PDT also generates substantial intracellular ROS, inducing LPO precipitation 56, ultimately resulting in the release of DAMPs 57. Additionally, the excessive ROS generated by PDT depletes glutathione (GSH) and inhibits GPX4 activity 58, which impairs the repairment of LPO damage, thereby sensitizing the ferroptotic effects. More importantly, PDT-induced endoplasmic reticulum stress potently promotes CRT translocation to the cell membrane, enhancing tumor cell immunogenicity 59. Collectively, PDT enhances the efficacy of tumor immunotherapy through multifaceted mechanisms.

Unlike the well-characterized mechanisms of immune checkpoint inhibitors, the role of anticoagulation therapy in immune modulation remains largely unexplored. For a long time, the coagulation system was perceived as functionally distinct from the immune system, despite the well-documented increased risk of thromboembolic events in cancer patients 60. However, emerging evidence has revealed intricate crosstalk between coagulation and immunity, challenging this traditional view. Cytokines, for instance, have been reported to stimulate leukocytes to express TF initiating the extrinsic coagulation cascades intravascularly 61. Meanwhile, the contact activation system bridges between the complement system and the intrinsic coagulation system 62. More importantly, platelets have been solidly recognized as an essential modulator of inflammatory responses 63, and recent studies have uncovered the new roles of platelets in immunosuppression in TME. First, platelets physically shield tumor cells from immune surveillance by forming a protective cloaking layer 26. Additionally, platelets release procancer growth factors, especially TGF-β to dampen functions of immune cells in the TME 64. Beyond platelets, thrombin has been implicated in driving immunosuppressive factors, like TGF-β 64. Furthermore, TF overexpressed in many types of cancers impairs T-cell effector functions 65. Finally, fibrin deposition in the TME creates a physical barrier that limits immune cell infiltration 66. Collectively, antiplatelets and anticoagulants may enhance antitumor immunity by disrupting the immunosuppressive mechanisms in the TME, which functions at the interface between PDT (induction of ICD) and immune checkpoint inhibitors (potentiating CTL attacks), thereby bridging the synergistic nature of three therapeutic modalities in immunotherapy.

## Conclusion

In summary, we developed a liposomal nanomedicine with PDIT effects (PDIT-liposome) by incorporating Pc (a photosensitizer to trigger PDT), rivaroxaban (a factor Xa inhibitor to promote T-cell priming), and αPD-L1 (a PD-L1 inhibitor to potentiate CTL attack). PDIT-liposome was fabricated with ROS-sensitive phospholipids, enabling the photo-induced disruption of liposomal scaffold and controlled drug release. Notably, the three components exerted immunostimulatory effects at distinct stages in immunotherapy, therefore augmenting the promotion of the overall immunotherapeutic efficacy. Moreover, the synergism with anticoagulation therapy and immune checkpoint inhibitors addresses the critical limitation of conventional PDT, lacking of systematic effects and persistence, by amplifying the immune responses invoked by PDT-induced ICD. Collectively, this study not only provides a highly efficient PDIT agent for oncotherapy but also establishes a theoretical foundation for the synergism of PDT, anticoagulation therapy, and immune checkpoint inhibition in oncotherapy.

## Supplementary Material

Supplementary figures and tables.

## Abbreviations

PDT: photodynamic therapy
PDIT: photodynamic immunotherapy
FXa: factor Xa
Pc: β-carbonylphthalocyanine
αPD-L1: a peptide inhibitor of PD-L1
P-liposome: liposomes containing Pc
RD-liposome: liposomes containing rivaroxaban and αPD-L1 peptide
PD-liposome: liposomes containing Pc and and αPD-L1 peptide
PR-liposome: liposomes containing Pc and rivaroxaban
PDIT-liposome: liposomes containing Pc, rivaroxaban and αPD-L1 peptide
ROS: reactive oxygen species
^1^O_2_: singlet-state oxygen
SEM: scanning electron microscopy
TEM: transmission electron microscopy
AFM: atomic force microscopy
HPLC: high-performance liquid chromatography
DLS: dynamic light scattering
PDI: polydispersity index
HD: hydrodynamic diameter
EE%: encapsulation efficiency
LE%: loading efficiency
CCK-8: Cell Counting Kit-8
DCFH-DA: 2', 7'-dichlorodihydro-fluorescein diacetate
DPBF: 2,5-diphenyl-2,4-benzofuran
SPR: Surface Plasmon Resonance
AM: Calcein-AM
PI: Propidium iodide
PD-L1: programmed death ligand-1
H&E: hematoxylin and eosin
IHC: immunohistochemistry
IF: immunofluorescence
DC: dendritic cell
ICD: immunogenic cell death
HMGB1: high mobility group box 1 protein
CRT: calreticulin
DAMPs: damage-associated molecular patterns
TME: tumor microenvironment
CTL: cytotoxic T lymphocyte
TDLNs: tumor-draining lymph nodes
