# Supplementary material for: ROS-sensitive liposomal co-delivery of photosensitizer, factor Xa inhibitor, and PD-L1 blockade enhances photodynamic immunotherapy
Source: Theranostics. 2026 Jan 21;16(8):4128–46. doi: 10.7150/thno.125408 (PMC12905798; doi:10.7150/thno.125408)
Supplement: Supplementary file 1 — Supplementary figures and tables. [file thnov16p4128s1.pdf]

Supplementary materials

ROS-sensitive liposomal co-delivery of photosensitizer, factor Xa inhibitor, and PD-L1 blockade enhances photodynamic immunotherapy

Yuhan Mai[1], Yanling Chen[2], Chao Li[1], Tongyao Wang[1], Shangli Ding[1], Hao Zhang[2], Haili Lin[3], Longguang Jiang[1], Cai Yuan[2,4], Xiaolei Zhou[*2], Mingdong Huang[*1], Peng Xu[*2,4]

[1] College of Chemistry, Fuzhou University, Fuzhou, Fujian 350116, China P.R.

[2] College of Biological Science and Engineering, Fuzhou University, Fuzhou, Fujian 350108, China P.R.

[3] Department of Pharmacy, The Peoples Hospital of Fujian Province, Fuzhou, Fujian 350004, China P.R.

[4] Fujian Key Laboratory of Marine Enzyme Engineering, Fuzhou University, Fuzhou, Fujian 350108, China P.R.

[*] Corresponding Authors (XL. Zhou, M. Huang and P. Xu)

E-mail: xiaolei.zhou@fzu.edu.cn; HMD_lab@fzu.edu.cn; pengxu@fzu.edu.cn

Postal address: Xueyuan Road, New College Town, Fuzhou, Fujian 350116, China

CONTENTS

1. Additional Experimental Procedures **4**

2. Supplementary Figures **9**

Figure S1 9

Figure S2 9

Figure S3 10

Figure S4 10

Figure S5 11

Figure S6 12

Figure S7 12

Figure S8 13

Figure S9 13

Figure S10 14

Figure S11 15

Figure S12 16

Figure S13 17

Figure S14 17

Figure S15 18

Figure S16 19

Figure S17 20

Figure S18 20

Figure S19 21

Figure S20 22

Figure S21 23

3. Supplementary Tables **24**

Table S1 24

Table S2 24

Table S3 25

Table S4 25

Table S5..25

Table S6..26

1. Additional Experimental Procedures

1.1 General

All chemicals were purchased from vendors and used without further purification unless specified. CT-26、EA.hy 926、LO2 cells were cultured in Dulbecco's Modified Eagle Medium (DMEM) supplemented with 10% fetal bovine serum (FBS), 0.5% streptomycin and 0.5% penicillin in an incubator with 5% CO_2 at 37 °C. Mice were housed together and maintained at a temperature of 25 °C with a 12-h light/dark cycle and supplied with sufficient food and water. All animal experiments complied with the National Research Council's Guide for the Care and Use of Laboratory Animals and were approved by the Animal Ethics Committee of the College of Biological Science and Engineering, Fuzhou University (2021-SG-072) and carried out in strict accordance with the guidelines.

1.2 Materials and reagents

Cell Counting Kit-8 (CCK-8), 2', 7'-dichlorodihydro-fluorescein diacetate (DCFH-DA) and 2,5-diphenyl-2,4-benzofuran (DPBF) were purchased from Sigma-Aldrich Co. Ltd. AM/PI living/dead cell double staining kit was purchased from Shanghai Beyotime Biotechnology Co., Ltd. Annexin V-FITC/PI apoptosis detection kit was purchased from Shanghai Yuanye Bio-Technology Co., Ltd.

1.3 Surface plasmon resonance (SPR) analysis

To determine the equilibrium binding constant (KD) of αPD-L1 peptide to PD-L1, surface plasmon resonance analysis (SPR) was conducted using a BIACORE T200 instrument (BIACORE, Uppsala, Sweden). The CM5 chip was coupled to PD-L1 by injecting 30 μg/mL of protein into immobilisation buffer (10 mM sodium acetate, pH 5.0) to achieve an immobilisation

level of approximately 10,000 response units (RUs). After immobilisation, the surface was closed with ethanolamine. The reference cell was prepared in the same manner but without coupling to PD-L1. Running buffer containing αPD-L1 peptide at a concentration of 100 μM was injected at a flow rate of 30 μL/min for 120 s at 25 °C. The cell was immobilised at a concentration of 1,000 RUs. Subsequently, dissociation was monitored for a duration of 300 seconds. The increased RU (delta RU) subtracted from the blank was recorded. Compounds with delta RU greater than 10 were selected to determine the K_D value. K_D values were determined using the above settings at a concentration range of 0-100 μM. K_D values were calculated based on steady-state-affinity modulation.

1.4 Determination of the reactive oxygen species (ROS) and singlet oxygen ($^1O_2$)

In the presence of ROS, DCFH-DA can be rapidly oxidized to strongly fluorescent 2',7'-dichlorofluorescein (DCF) derivatives. The different liposome groups were mixed with 25 μM DCFH-DA, respectively. The solution was illuminated with near-infrared light irradiation (680 nm, 45 mW/cm^2), and then the fluorescence of the probe was tracked (Ex: 488 nm, Em: 525 nm).

The ability of the PDIT-liposome to generate $^1O_2$ was measured by monitoring the change in absorbance of DPBF at 415 nm under irradiation (680 nm, 45 mW/cm^2).

1.5 Determination of the enzymatic kinetics of FXa to verify rivaroxaban's inhibitory potency

To verify that rivaroxaban was released after light trigger of the liposomes and still had factor X inhibitory activity, the enzyme activity profile of the released solution against factor X was determined. The liposome suspension (5 mL) was taken and placed into a dialysis membrane (MWCO 1.5 kDa), which was placed into a PBS solution filled with 5 mL of PBS containing

Tween-80 (pH 7.4, n = 3), and the dialysis tubing was incubated in the dark at 37 °C with agitation (50 rpm). After irradiation with a LED light source (680 nm, 40.5 J/cm^2), 20 µl of dialysate was taken and 50 µl buffer (30 mM Tris, 150 mM NaCl pH=7.4), 10 µl $CaCl_2$ (50 mM),10 µl FXa (activated Human Factor X, 1.5 µM) were added and incubated at room temperature on a shaking table After 15 min, 10 µl of S-2765 (Factor Xa chromogenic substrate, 5 mM) was added; 405 nm UV absorption was recorded once every 30 s for 15 min. The buffer group was set as the blank control group, and rivaroxaban (0.1 µM) was set as the positive control group.

1.6 Cell Culture, Cytotoxicity and Biosafety of PDIT-liposome

HCT-116, CT-26, EA.hy 926, and LO2 (ATCC) cells were cultivated in Dulbecco's Modified Eagle Medium (DMEM) supplemented with 10% FBS, 0.5% streptomycin and penicillin at 37 °C under 5% CO_2. PDIT-liposome cytotoxicity was assessed via CCK-8 assay: (1) Time-dependent assay—HCT-116 cells were treated with 2 mg/mL liposomes (0–24 h) with (phototoxicity) or without (dark toxicity) NIR irradiation (680 nm, 1.5 J/cm^2), and (2) Dose-dependent assay—HCT-116 cells were exposed to 0-2 mg/mL liposomes (6 h) with (phototoxicity) or without (dark toxicity) light irradiation (680nm). Cell viability was evaluated by measuring A450 values. PDIT-liposome biosafety was evaluated by hemolysis testing in erythrocytes and cytotoxicity in EA.hy 926/LO2 cells. Briefly, Erythrocytes from healthy ICR mice were washed, diluted with saline, and treated with blank control liposome and PDIT-liposome (500 µL of 2% erythrocytes) for 30 min at 37 °C. Saline and ds-water served as negative and positive controls, respectively. After incubation, the supernatants were centrifuged (2000 rpm, 2 min), and A540 was measured. The hemolytic rate was calculated as: Hemolysis =

$(OD_{sample}–OD_{blank\ control})/(OD_{positive\ control}–OD_{blank\ control})$. The cytotoxicity of liposomes against endothelial EA.hy 926 and human liver LO2 cell lines was evaluated by the CCK-8 and Live/Dead staining assay with Calcein-AM/PI as previously described. To evaluate the effects of PDIT liposomes on the coagulation system, the activated partial thromboplastin time (APTT) and prothrombin time (PT) were measured in BALB/c mice (n=5 per group) after tail vein injection of control liposomes, PDIT liposomes, or saline (2 mg/kg) in 24 h. Additionally, blood biochemistry analysis evaluated liver function markers (ALT, AST, and ALP), renal function markers (BUN and Crea), and metabolic markers (UA) after tail vein injection of control liposomes, PDIT liposomes, or saline (2 mg/kg) in 9 h.

1.7 Live/dead staining

HCT-116 cells were inoculated into 96-well plates at a density of 5×10^3 cells per well and cultured for 24 h. The cells were then treated with 2 mg/mL of liposomes for 6 h, respectively. The light group was irradiated by near-infrared light irradiation (680 nm, 1.5 J/cm^2). Next, the cells were stained using Calcein-AM (AM) and propidium iodide (PI) double staining kit. Finally, images of live and dead cells were collected by high content analysis system(Operetta CLS, PerkinElmer, Waltham, MA).

1.8 Intracellular ROS generation

HCT-116 cells were inoculated into 96-well plates at a density of 5×10^3 cells per well and cultured for 24 h. The cells were then treated with 2 mg/mL of liposomes for 6 h, respectively. After DCFH-DA staining for 0.5 h, the cells were washed with PBS. The light group was irradiated by near-infrared light irradiation (680 nm, 1.5 J/cm^2). Finally, cell images were collected using a high-content analysis system.

1.9 Apoptosis and necroptosis imaging

HCT-116 cells were inoculated into 96-well plates at a density of 5×10^3 cells per well and cultured for 24 h. The cells were then treated with 2 mg/mL of liposomes for 6 h, respectively. The light group was irradiated by near-infrared light irradiation (680 nm, 1.5 J/cm^2). Next, cells were stained using the Annexin V-FITC/Propidium Iodide (PI) Apoptosis Detection Kit following the manufacturer's instructions. Finally, cell images were collected using a high content analysis system.

1.10 Evaluation of synergistic effects by the Bliss model

In the Bliss model, E_P, E_{PD}, E_{PR}, and E_{PDIT} represent the antitumor rates of the P-liposome, PD-liposome, PR-liposome, and PDIT-liposome groups, respectively. Determination of synergistic effects through calculation and comparison of the Extra Addition Predicted Value (E_{add}), Bliss Independent Effect Predicted Value (E_{bliss}), and the actual observed data (E_{PDIT}): Additional simple summation predicted value (Eadd), Bliss independent effect predicted value (E_{bliss}), and actual observed data (E_{PDIT}): 1) E_{add}: If the effects of Pc, rivaroxaban, and αPD-L1 peptide are simply additive, then $E_{add} = E_{PD} + E_{PR} - E_P$; 2) E_{bliss}: Under the Bliss model assumption that components do not interfere with each other (independence), the combined additional effect of rivaroxaban and αPD-L1 peptide based on Pc is: $\Delta E_{PR\_bliss} = (E_{PD} - E_P) + (E_{PR} - E_P) - (E_{PD} - E_P)(E_{PR} - E_P)$. Then, $E_{blis}s = E_P + \Delta E_{PR\_bliss}$; 3) Evaluation of Synergy: If $E_{PDIT} > E_{add}$, it indicates therapeutic effects exceeding simple addition, demonstrating synergistic effects; if $E_{PDIT} > E_{bliss}$, it signifies therapeutic effects surpassing the independent effect expectation, indicating strong synergistic effects.

2. Supplementary Figures

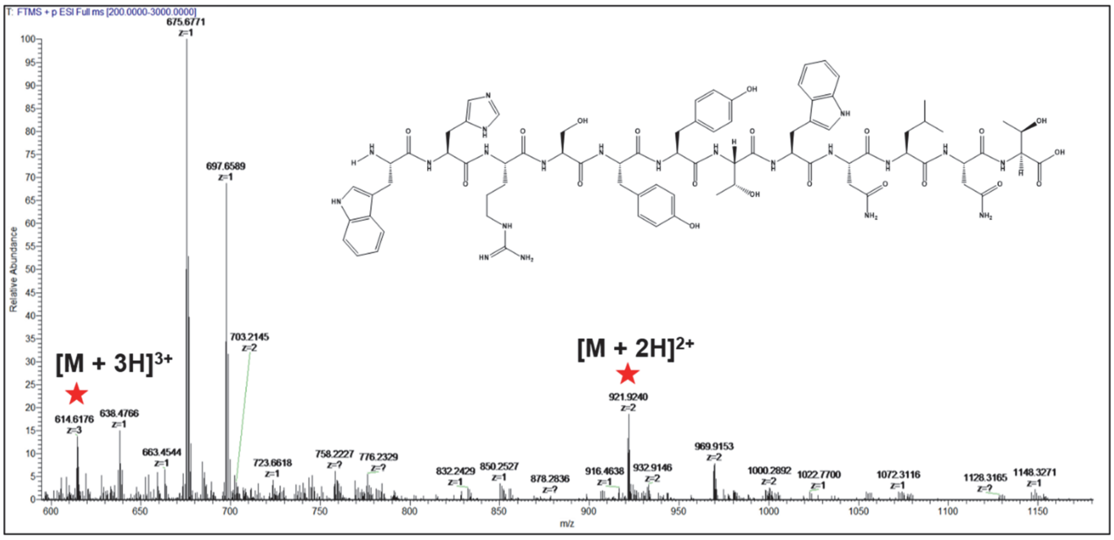

Figure S1. HRMS spectrum of αPD-L1 peptide.

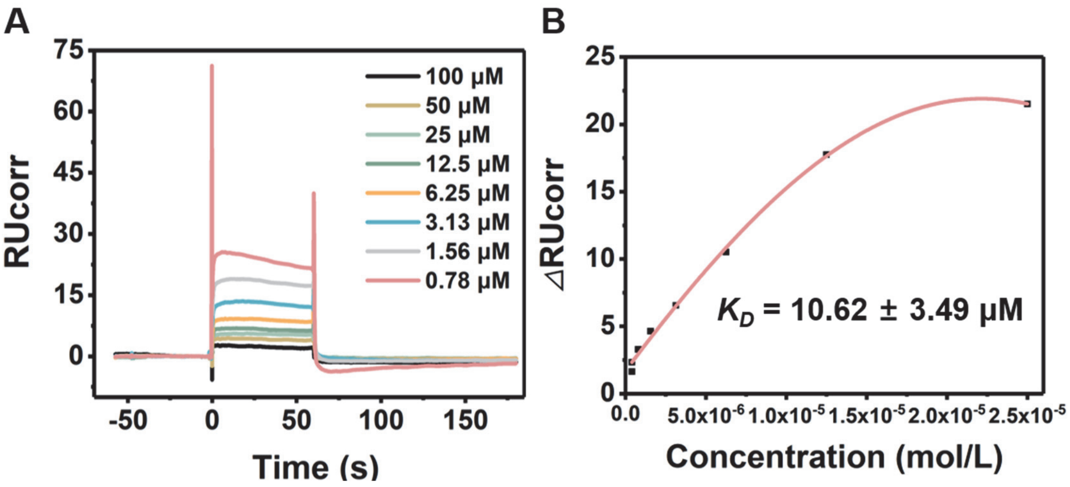

Figure S2. The binding ability of αPD-L1 peptide to PD-L1 was measured by SPR(surface plasmon resonance) assay. Recombinant αPD-L1peptide flowed through a CM5 chip immobilized with PD-L1, αPD-L1 peptide concentrations were from top to bottom 100, 50, 25, 12.5, 6.25, 3.13, 1.56, 0.78 μM.

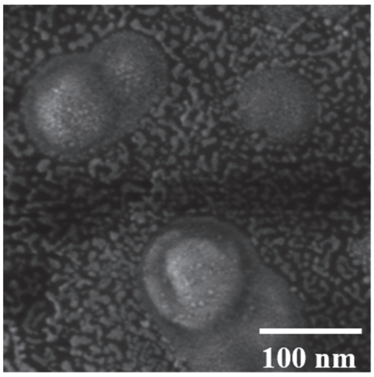

Figure S3. SEM image of PDIT-liposome.

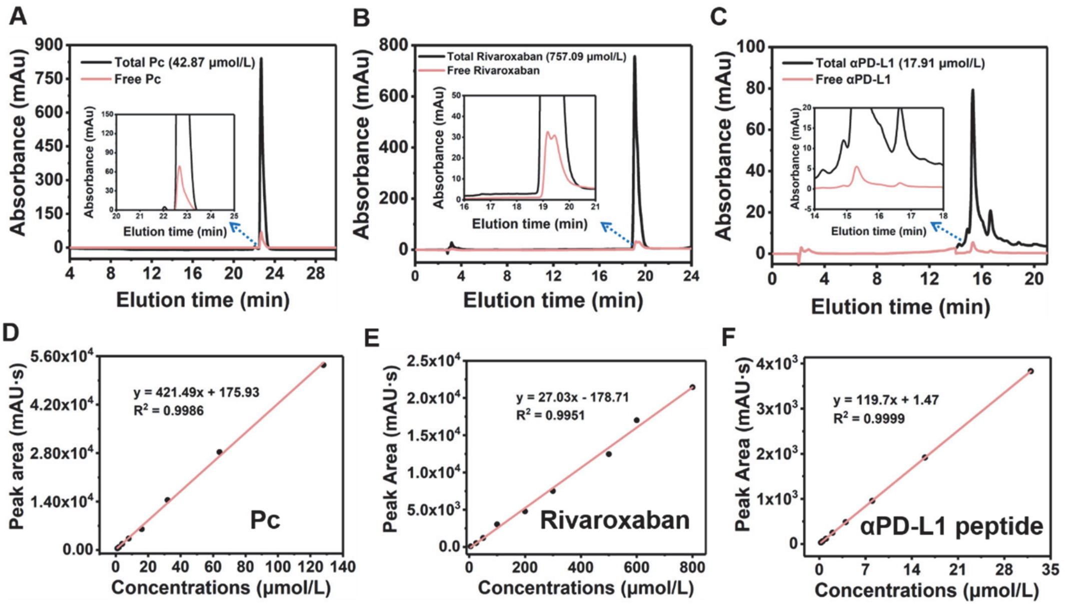

Figure S4. Evaluation of average encapsulation efficiency of Pc(A)、rivaroxaban(B) and αPD-L1 peptide(C) by HPLC. Linear fitting curve of HPLC peak area against the concentration of Pc(D)、rivaroxaban(E) and αPD-L1 peptide(F).

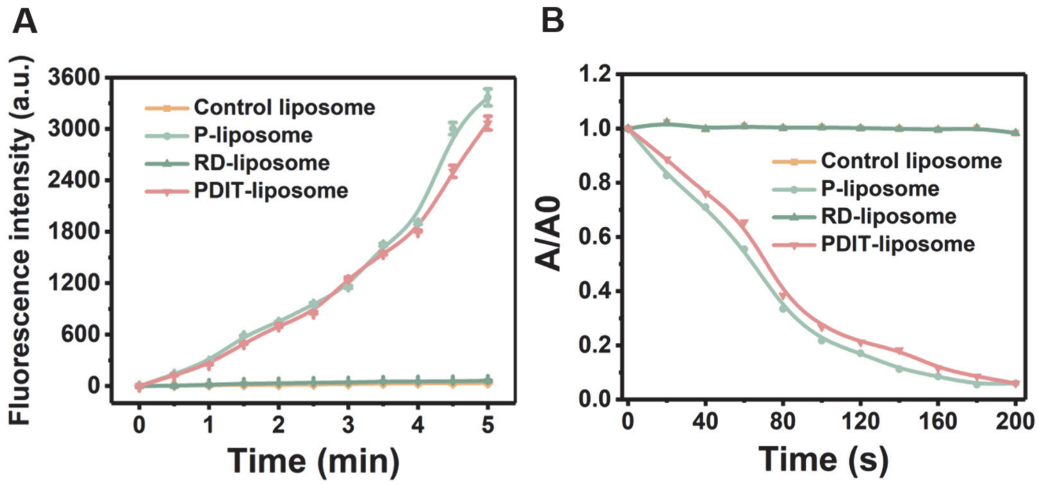

Figure S5. Photodynamic effect of PDIT-liposome. (A) Light-induced production of ROS by PDIT-liposome using DCFH-DA as a probe; (B) Absorbance variations of DPBF at 415 nm treated with PDIT-liposome under irradiation by a 680 nm diode laser for different times.

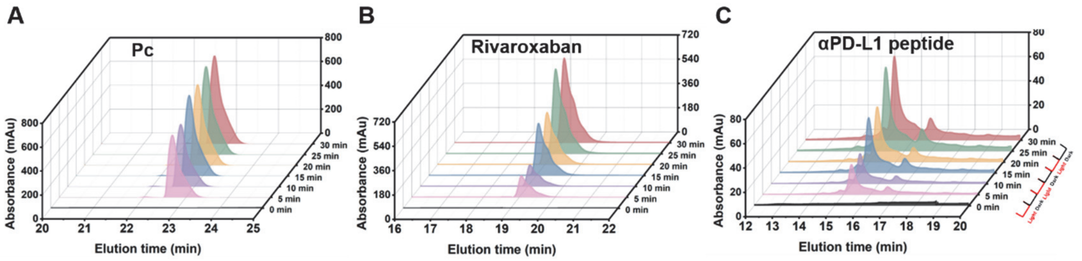

Figure S6. Photostimulated liposomes to release Pc(A)、rivaroxaban(B) and αPD-L1 peptide(C) were determined in dark conditions and upon light irradiation (680 nm, 45 mW/cm^2) at identical time points by HPLC.

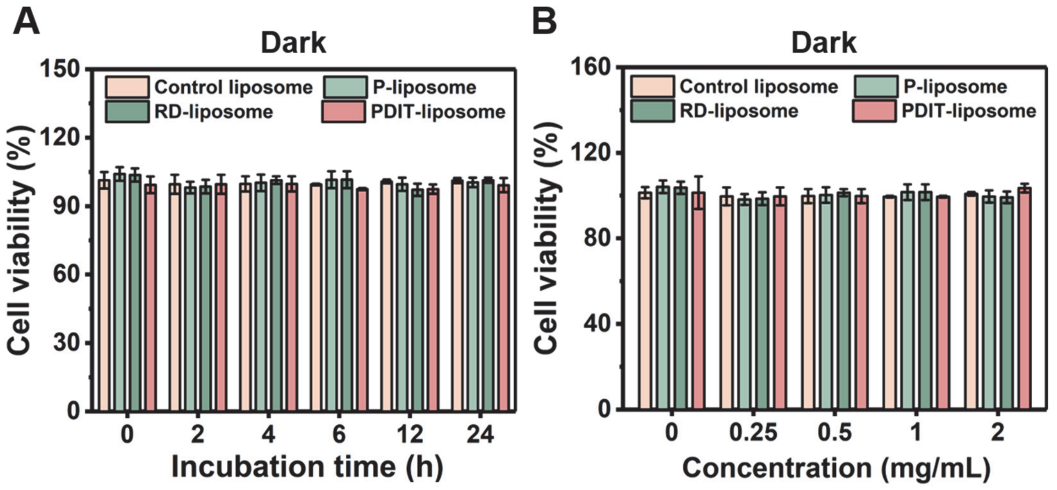

Figure S7. Dark cytotoxicity of PDIT-liposome *in vitro*. (A) Incubation time-dependent (A, 2 mg/mL) and drug dose-dependent (B, 6 h) cytotoxicity of control liposome, P-liposome, RD-liposome, and PDIT-liposome against HCT-116 cells

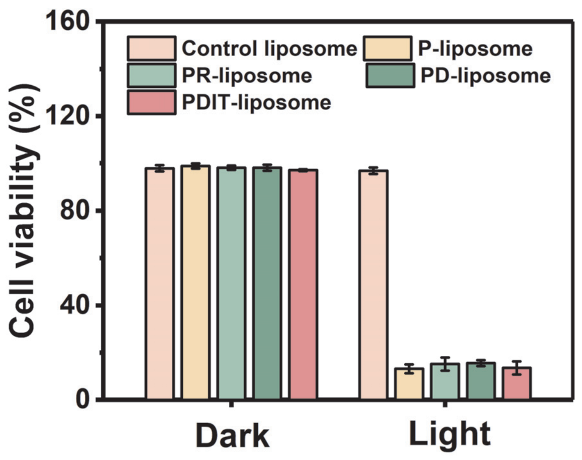

Figure S8. Cytotoxicity of control liposome, P-liposome, PR-liposome, PD-liposome, and PDIT-liposome against HCT-116 cells with 6 h of incubation time and 2 mg/mL of drug dose. Photocytotoxicity was triggered by irradiation with a 680 nm LED light source (1.5 J/cm^2).

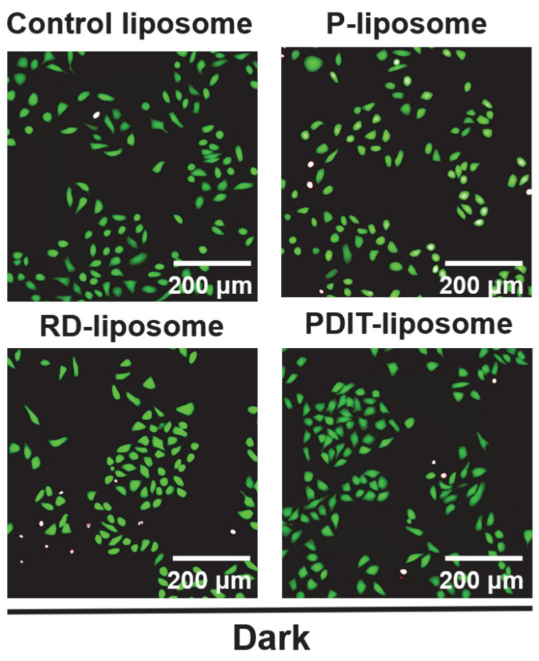

Figure S9. Representative live/dead staining images of HCT-116 cells treated with various liposomes in dark. Live and dead cells were fluorescently imaged by Calcein-AM (green, ex480/em500) and PI (red, ex490/em635), respectively.

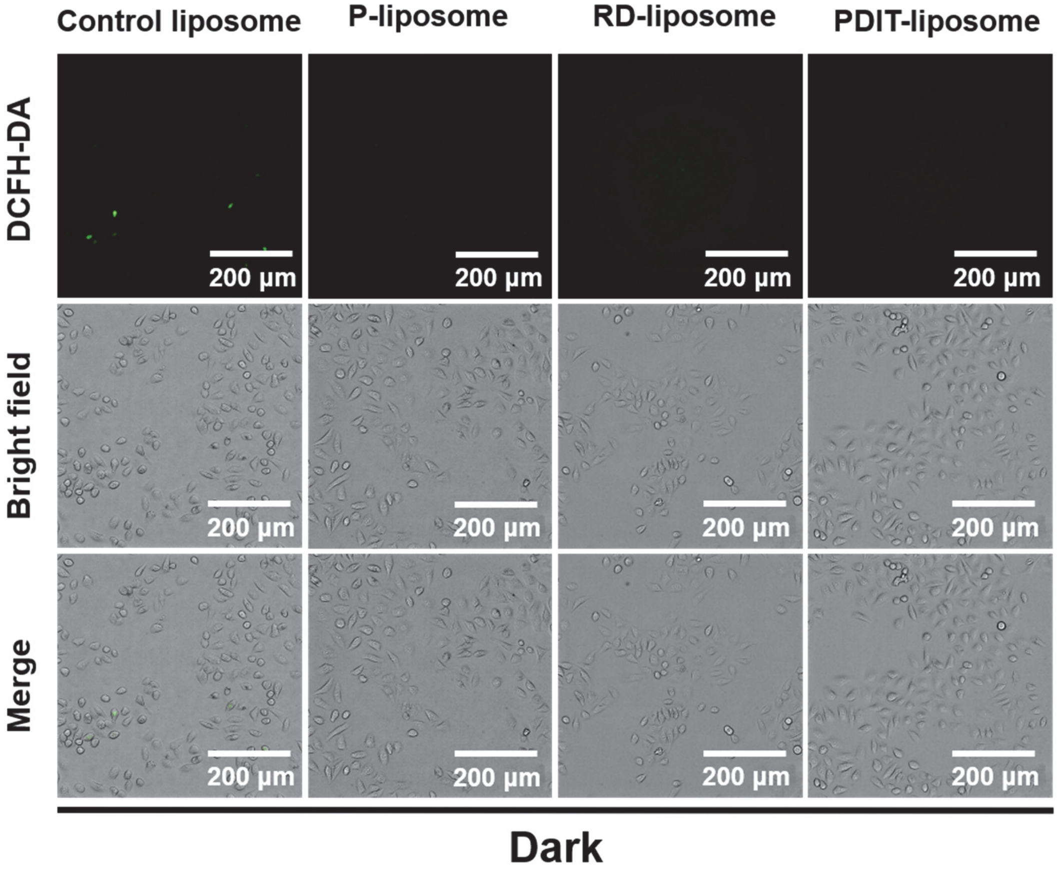

Figure S10. Intracellular ROS generation by PDIT-liposome. ROS was imaged with DCFH-DA as the fluorescent probe. HCT-116 cells treated with various liposomes in dark.

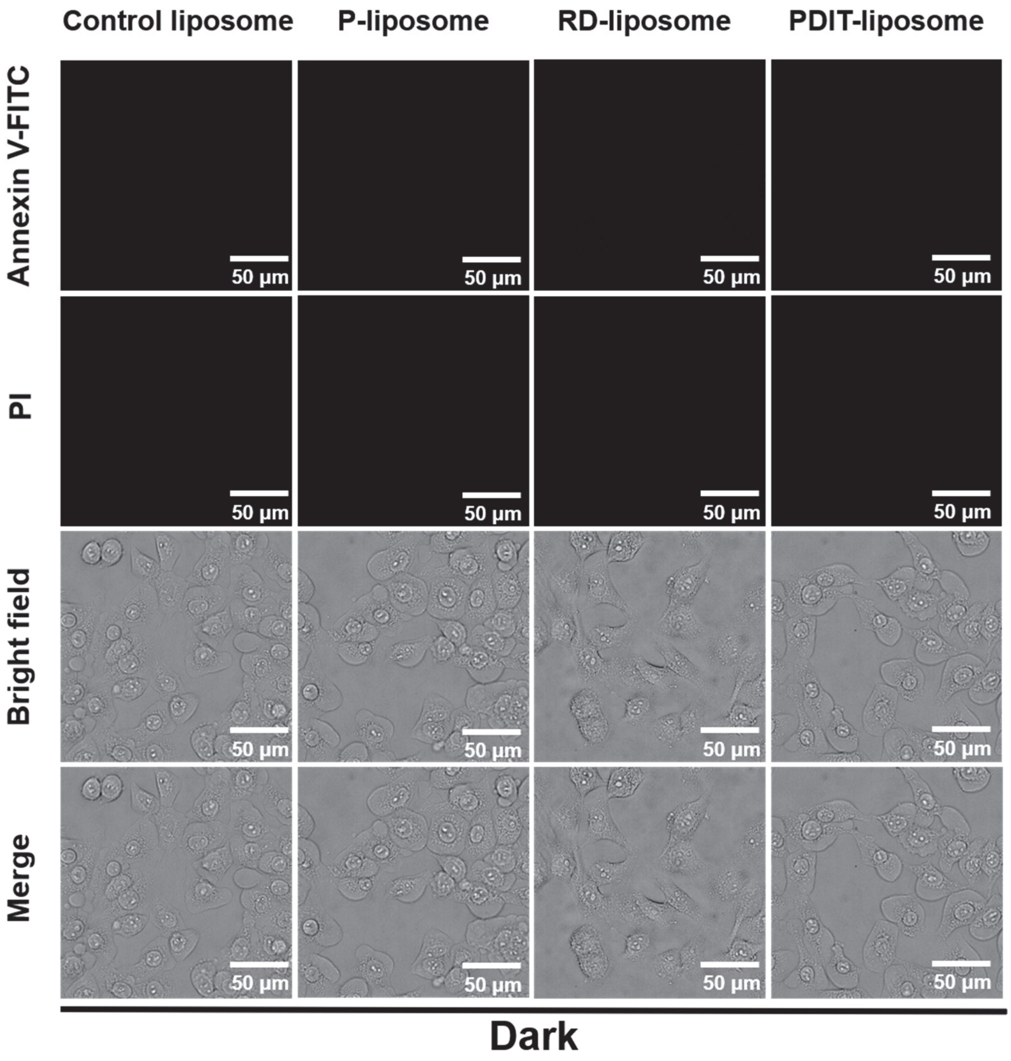

Figure S11. Fluorescent imaging of apoptotic and necroptotic HCT-116 cells stained with Annexin V-FITC (green, ex494/em518) and PI (red, ex490/em635). HCT-116 cells treated with various liposomes in dark.

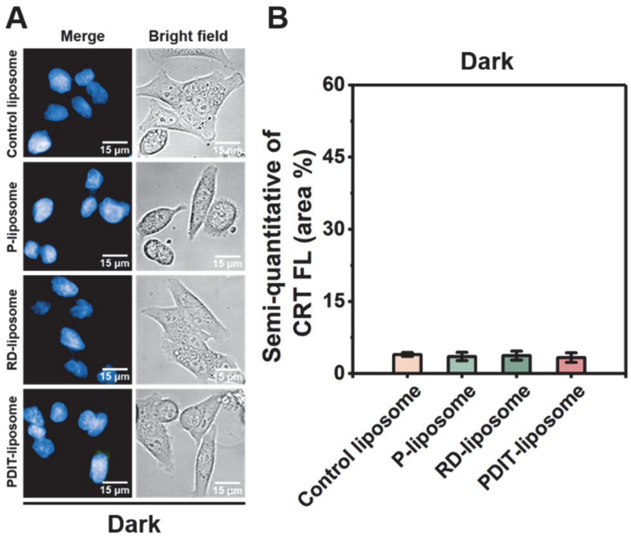

Figure S12. (A) Fluorescent imaging of CRT exposure on the surface of HCT-116 cells stained with hoechest 33342 (blue, ex350/em460) and ecto-CRT targeted imaging probe, CREpep-FITC (green, ex485/em538). Cells were treated with various liposomes (2 mg/mL) for 6 h. (B) Corresponding fluorescence intensity in cells was semi-quantitatively calculated using ImageJ software.

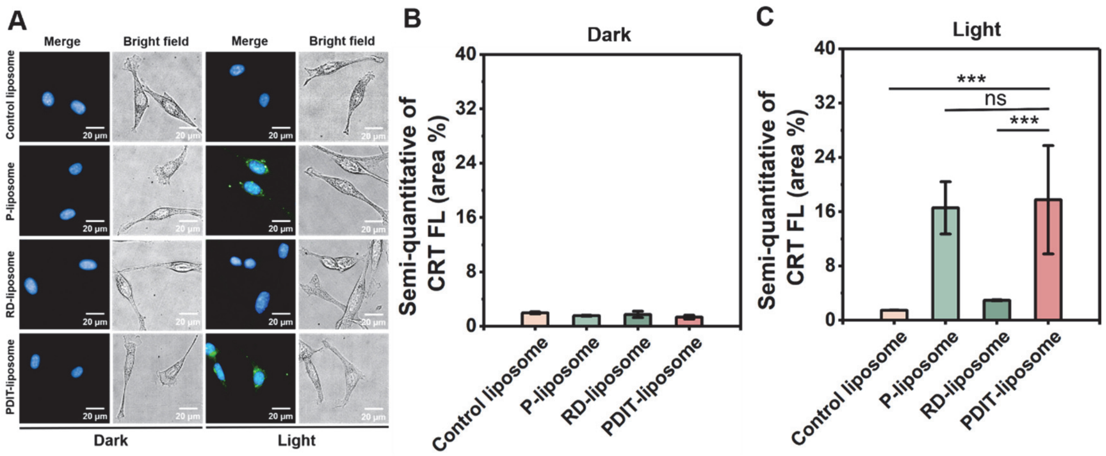

Figure S13. (A) Fluorescent imaging of CRT exposure on the surface of CT-26 cells stained with hoechest 33342 (blue, ex350/em460) and ecto-CRT targeted imaging probe, CREpep-FITC (green, ex485/em538). Cells were treated with various liposomes (2 mg/mL) for 6 h, followed by maintenance in dark (B) or irradiation (C, 680nm, 1.5 J/cm^2), (B-C) Corresponding fluorescence intensity in cells without (B) or with (C) irradiation was semi-quantitatively calculated using ImageJ software.

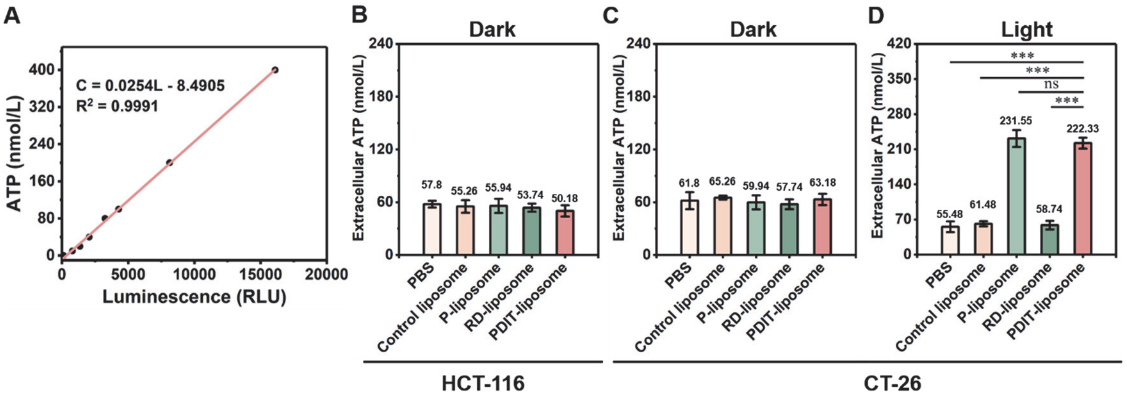

Figure S14. (A) The standard curve of ATP. (B) Extracellular secretion of ATP in HCT-116 cells in dark (B) and in CT-26 cells in dark (C) or after irradiation (D, 680nm, 1.5 J/cm^2). Data are presented as Mean ± SD (n = 6, **P <0.01, ***P < 0.001).

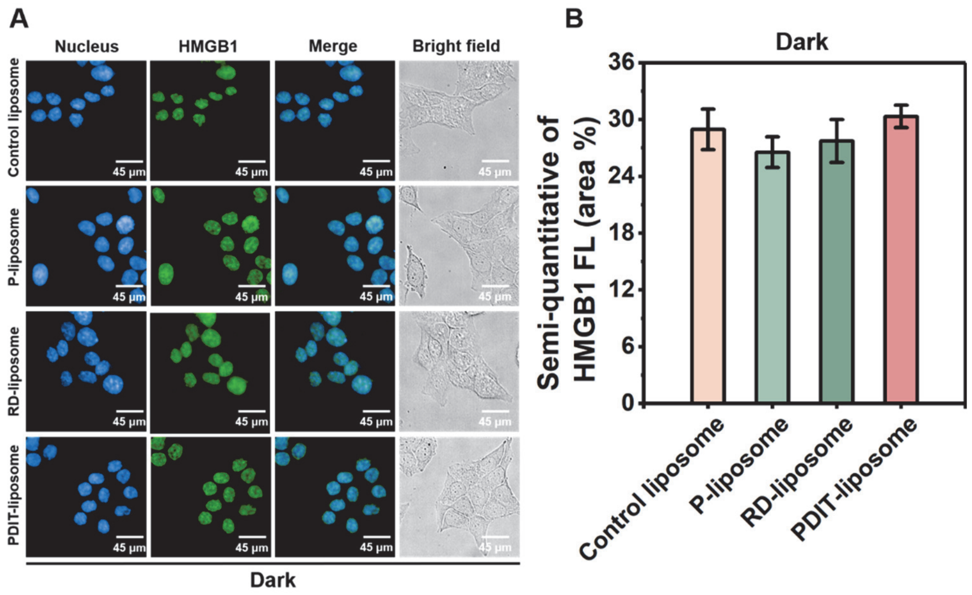

Figure S15. (A) Fluorescent imaging of HMGB1 release from HCT-116 cells stained with hoechest 33342 (blue, ex350/em460), primary antibodies against HMGB1 and Alexa Fluor 594-conjugated secondary antibody (green, ex594/em617). Cells were treated with various liposomes (2 mg/mL) for 6 h before imaging. (B) Corresponding fluorescence intensity was semi-quantitatively calculated using ImageJ software.

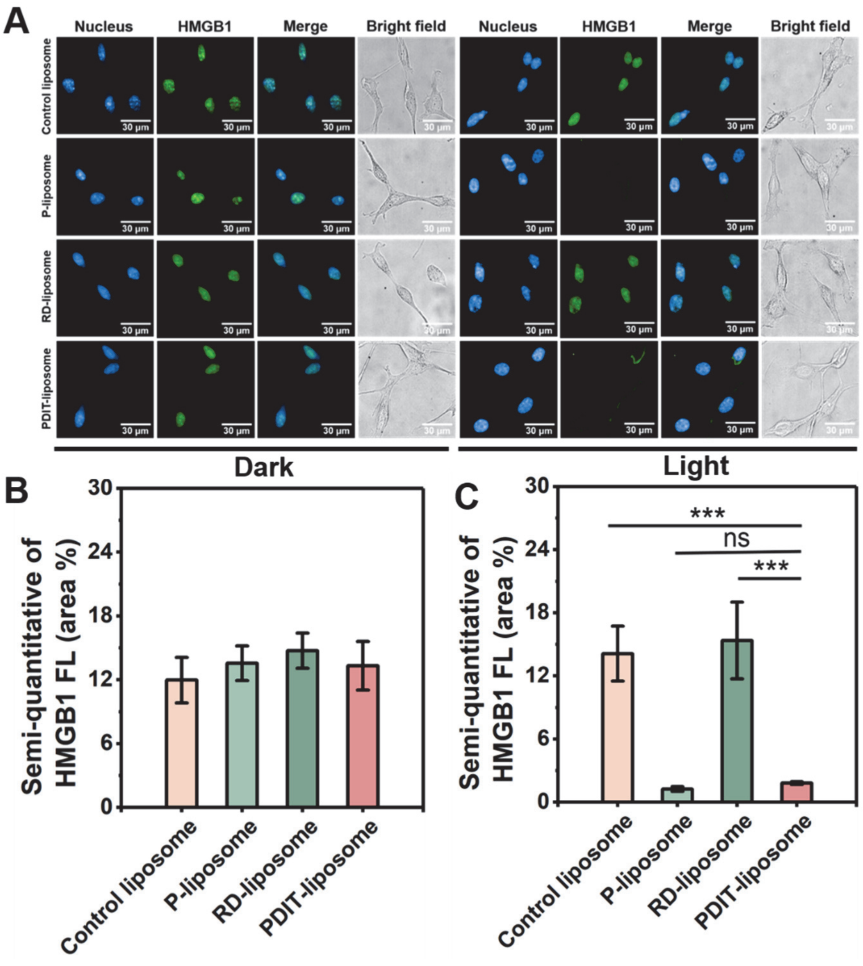

Figure S16. (A) Fluorescent imaging of HMGB1 release from CT-26 cells stained with hoechest 33342 (blue, ex350/em460), primary antibodies against HMGB1 and Alexa Fluor 594-conjugated secondary antibody (green, ex594/em617). Cells were treated with various liposomes (2 mg/mL) for 6 h, followed by maintenance in dark (B) or irradiation (C, 680nm, 1.5 J/cm^2) or before imaging. (B-C) Corresponding fluorescence intensity in cells without (B) or with (C) irradiation was semi-quantitatively calculated using ImageJ software. Data are presented as Mean ± SD (n = 6, **P < 0.01, ***P < 0.001).

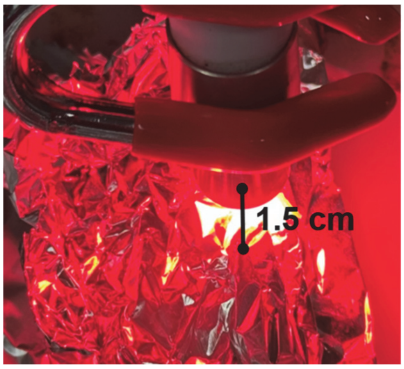

Figure S17. Photographs of operation for light radiation in tumor site.

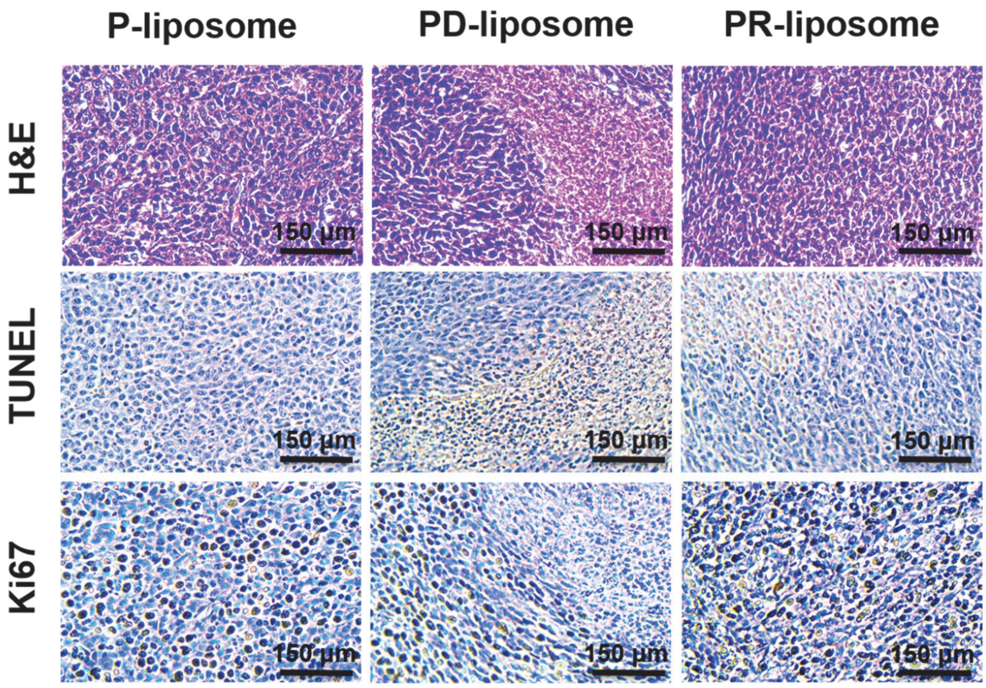

Figure S18. Hispopathological sections of tumor tissues from P-liposome, PD-liposome and PR-liposome groups were stained with H&E, TUNEL, respectively.

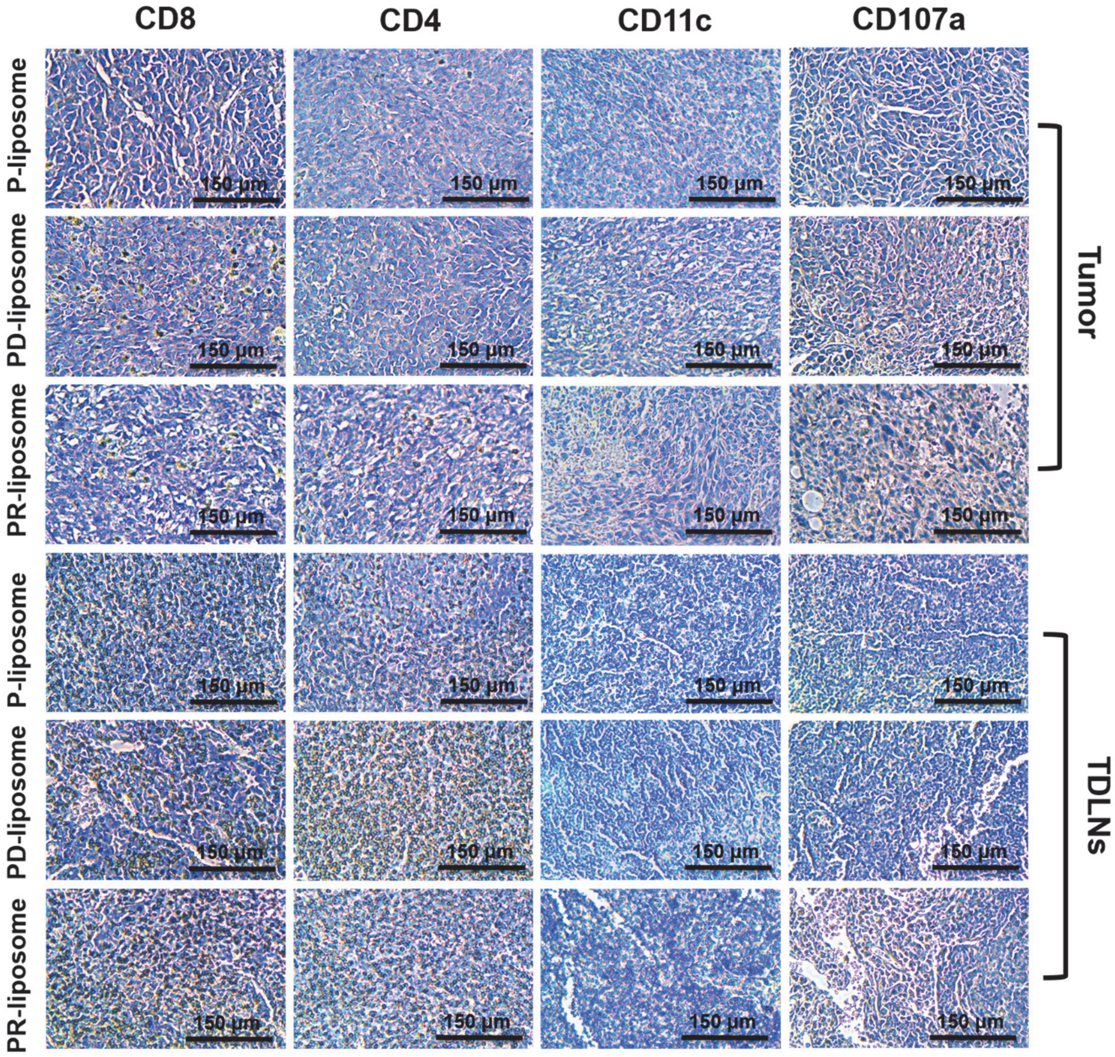

Figure S19. Hispopathological sections of tumor tissues and TDLNs from P-liposome, PD-liposome and PR-liposome groups were stained with CD8, CD4, CD107a, and CD11c, respectively.

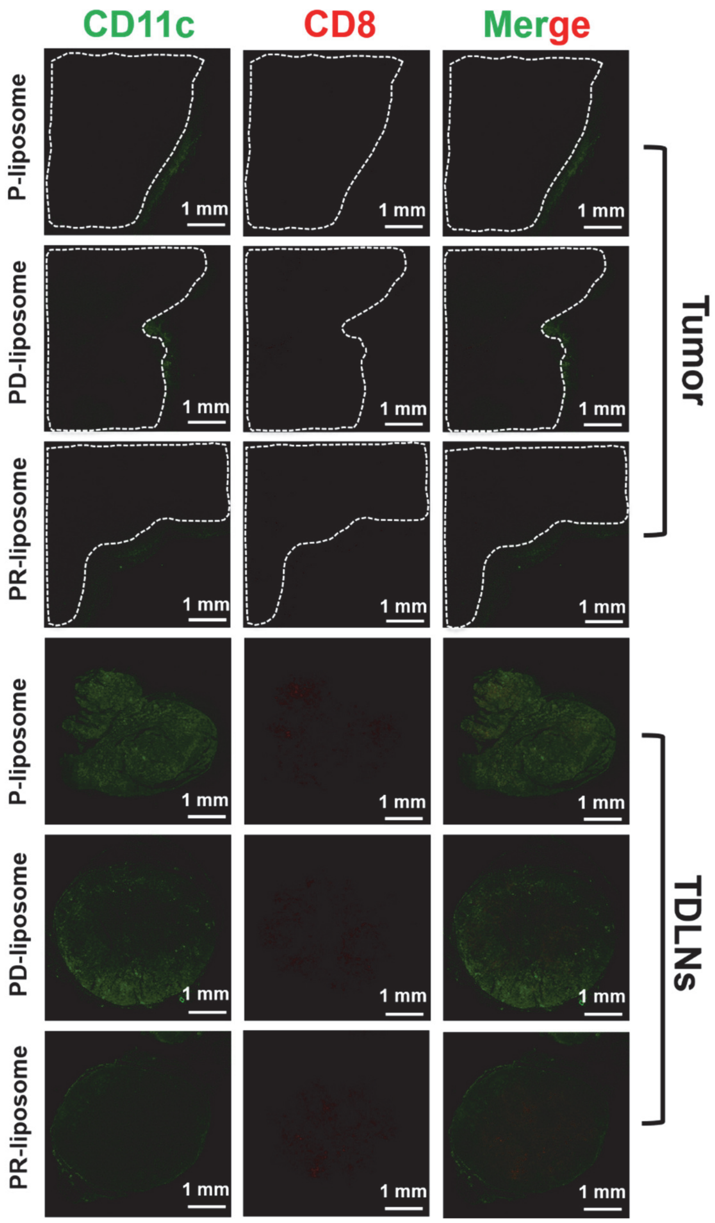

Figure S20. Immunofluorescent analysis of tumor and TDLNs from P-liposome, PD-liposome and PR-liposome groups were double stained with CD8 and CD11c, respectively. The white dashed coil represents the tumor region and yellow merged fluorescence represents the $CD8^{+}CD11c^{+}$ positive DCs.

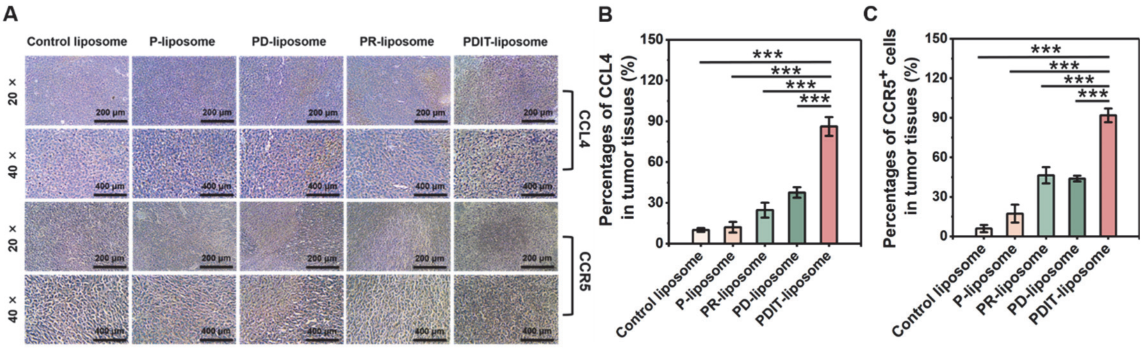

Figure S21. (A) Representative histopathological sections of tumor tissues stained with CCL4 and CCR5, respectively. (B-C) Quantitation of CCL4$^+$ cells (B) and CCR5$^+$ cells (C) in tumor sections. Data are presented as Mean ± SD (n = 6, **P < 0.01, ***P < 0.001).

3. Supplementary Tables

Table S1. Size of liposomes measured by different methods (The results of DLS represents hydrated particle size, The results of TEM、SEM、AFM represents electron microscope particle size).

Type of particle size		Sample 1	Sample 2	Sample 3	Mean±SD
**Hydrated particle size**	**DLS**	104.2	108.8	95.87	102.96 ± 6.55
**Electron microscope particle size**	**TEM**	123.4	138.4	128.7	130.17 ± 7.61
	**SEM**	94.99	118.7	90.95	101.55 ± 14.99
	**AFM**	106.82	108.47	115.04	110.11 ± 4.35

Table S2. Size、PDI of liposomes and EE% of each Pc、rivaroxaban and αPD-L1 peptide.

	Size	PDI	EE (%) of Pc	EE (%) of rivaroxaban	EE (%) of αPD-L1
**1st batch**	104.2	0.164	91.83	93.67	95.55
**2nd batch**	108.8	0.115	93.28	94.67	96.1
**3rd batch**	95.87	0.128	92.72	93.56	95.38
**Mean±SD**	102.96 ± 6.55	0.136 ± 0.02	92.61 ± 0.73	93.97 ± 0.61	95.68 ± 0.38

Table S3. LE% of Pc、rivaroxaban and αPD-L1 peptide in PDIT-liposome.

	LE (%) of Pc	LE (%) of rivaroxaban	LE (%) of αPD-L1
**1st batch**	0.56	7.18	0.73
**2nd batch**	0.57	7.26	0.74
**3rd batch**	0.56	7.17	0.73
**Mean±SD**	0.56 ± 0.006	7.2 ± 0.049	0.73 ± 0.006

Table S4. Evaluation of blood biochemistry (n = 5), including ALT、AST、ALP、BUN、CREA and UA.

	ALT (U/L)	AST (U/L)	ALP (U/L)	BUN (mg/dl)	CREA (μmol/L)	UA (μmol/L)
**Saline**	37.76 ± 4.65	67.75 ± 7.45	59.31 ± 7.54	26.3 ± 3.98	22.96 ± 4.17	109.48 ± 13.09
**Control liposome**	36.6 ± 3.56	68.19 ± 6.95	59.53 ± 10.28	25.48 ± 2.92	22.26 ± 1.19	110.94 ± 10.11
**PDIT-liposome**	36.58 ± 2.14	67.79 ± 5.18	60.89 ± 6.16	26.14 ± 2.79	23.41 ± 1.65	110.45 ± 12.89

Table S5. APTT and PT of plasma samples from mice administrated with saline, control liposome, or PDIT-liposome (2 mg/kg).

	APTT (s)	PT (s)
**Saline**	28.51 ± 1.36	12.01 ± 1.22
**Control liposome**	28.05 ± 2.32	11.18 ± 1.35
**PDIT-liposome**	27.37 ± 3.06	12.83 ± 1.75

Table S6. Additional simple summation predicted value (E_{add}) and Bliss independent effect predicted value (E_{bliss}) calculated by the Bliss model.

E_P	E_{PD}	E_{PR}	E_{PDIT}	E_{add}	E_{bliss}
21.83%	46.78%	51.08%	91.7%	76.03%	68.73%
